# The *cadDX* operon contributes to cadmium resistance, oxidative stress resistance, and virulence in zoonotic streptococci

**DOI:** 10.1186/s13567-024-01371-1

**Published:** 2024-09-27

**Authors:** Xinchi Zhu, Zijing Liang, Jiale Ma, Jinhu Huang, Liping Wang, Huochun Yao, Zongfu Wu

**Affiliations:** 1 MOE Joint International Research Laboratory of Animal Health and Food Safety, College of Veterinary Medicine, Nanjing Agricultural University, Nanjing, 210014 China; 2 Key Lab of Animal Bacteriology, Ministry of Agriculture, Nanjing, 210014 China; 3 WOAH Reference Lab for Swine Streptococcosis, Nanjing, 210014 China; 4 Guangdong Provincial Key Laboratory of Research On the Technology of Pig Breeding and Pig Disease Prevention, Guangzhou, 511400 China

**Keywords:** *Streptococcus suis*, *Streptococcus agalactiae*, mobile genetic elements, oxidative stress response, cadmium resistance, bacterial virulence

## Abstract

Mobile genetic elements (MGEs) enable bacteria to acquire novel genes and traits. However, the functions of cargo genes within MGEs remain poorly understood. The cadmium resistance operon *cadDX* is present in many gram-positive bacteria. Although *cadDX* has been reported to be involved in metal detoxification, its regulatory mechanisms and functions in bacterial pathogenesis are poorly understood. This study revealed that *cadDX* contributes to cadmium resistance, oxidative stress resistance, and virulence in *Streptococcus suis*, an important zoonotic pathogen in pigs and humans. CadX represses *cadD* expression by binding to the *cadDX* promoter. Notably, *cadX* responds to H_2_O_2_ stress through an additional promoter within the *cadDX* operon*,* mitigating the harmful effect of excessive *cadD* expression during oxidative stress. *cadDX* resides within an 11 K integrative and mobilizable element that can autonomously form circular structures. Moreover, *cadDX* is found in diverse MGEs, accounting for its widespread distribution across various bacteria, especially among pathogenic streptococci. Transferring *cadDX* into another zoonotic pathogen, *Streptococcus agalactiae*, results in similar phenotypes, including resistance to cadmium and oxidative stresses and increased virulence of *S. agalactiae* in mice. The new functions and regulatory mechanisms of *cadDX* shed light on the importance of the *cadDX* system in driving evolutionary adaptations and survival strategies across diverse gram-positive bacteria.

**Supplementary Information:**

The online version contains supplementary material available at 10.1186/s13567-024-01371-1.

## Introduction

Bacteria can acquire new genes via mobile genetic elements (MGEs), conferring additional attributes, including resistance to antibiotics and heavy metals, toxin production, and the ability to metabolize a wide range of compounds [1–4]. This process enables bacteria to adapt quickly to environmental stresses and promotes evolutionary innovation [5, 6]. The cadmium resistance operon *cadDX* comprises a P-type ATPase CadD and a member of the ArsR family transcriptional repressor CadX, which is crucial in conferring cadmium resistance in gram-positive bacteria. *cadDX* has been reported to be a plasmid-borne system in various bacteria [7–11]. In *Staphylococcus aureus* pI258, CadD functions as an energy-dependent efflux pump, increasing the minimum inhibitory concentration (MIC) of cadmium (Cd^2+^) by approximately 1000-fold [8], whereas CadX regulates the transcription of *cadD* by binding to its promoter [11]. In the plasmid pLUG10 of *Staphylococcus lugdunensis*, a mutation in either *cadD* (also called *cadB*) or *cadX* reduces cadmium resistance, indicating that both genes are required for full cadmium resistance [7]. In *Streptococcus salivarius* 57. I, *cadD* is responsible for resistance to Cd^2+^ and zinc (Zn^2+^), whereas CadX senses Cd^2+^ or Zn^2+^ and negatively regulates *cadD* expression [12]. A recent study on *Streptococcus agalactiae* reported that *cadD* is involved in the detoxification of metals, such as Zn^2+^, Cd^2+^, copper (Cu^2+^), cobalt (Co^2+^), and nickel (Ni^2+^), and promotes immune evasion and bacterial colonization in pregnant hosts [13]. However, the regulatory mechanisms and functions of *cadDX* in bacterial pathogenesis are poorly understood.

*Streptococcus suis* is a pathogen capable of causing systemic diseases such as septicemia and meningitis in pigs [14]. It is also considered a zoonotic pathogen, posing a risk for humans with close contact with infected pigs or contaminated by-products [15, 16]. In this study, we revealed that *cadDX* contributes to cadmium resistance, oxidative stress resistance, and virulence in *S. suis*. Notably, *cadX* possesses its own promoter and promotes oxidative stress by preventing excessive expression of *cadD*, which harms *S. suis* survival during oxidative stress. Additionally, *cadDX* exists within an 11 K integrative and mobilizable element (IME) that can autonomously form circular structures in *S. suis*. Furthermore, we found that *cadDX* also confers resistance to cadmium and oxidative stresses and enhances the virulence of *S. agalactiae*, extending its phenotypic effects to different bacterial hosts.

## Materials and methods

### Bacterial strains and culture conditions

All strains and plasmids used in this study are listed in Additional file 1. *S. suis* and *S. agalactiae* strains were cultured in Todd-Hewitt broth (THB, Becton Dickinson, USA) and plated on THB agar (THA) medium containing 6% (vol/vol) sheep blood at 37 °C with 5% CO_2_. *Escherichia coli* strains were grown in Luria–Bertani (LB; Becton Dickinson, USA) broth at 37 °C. The following antibiotics were added as needed: spectinomycin (Macklin, China) at 50 μg/mL for *E. coli,* 100 μg/mL for *S. suis*, and kanamycin (Macklin, China) at 50 μg/mL for *E. coli*. The metal salts, including ZnCl_2_, CdCl_2_, CuSO_4_, MnCl_2_, MgCl_2_, or NiCl_2_ (Sinopharm Chemical Reagent Co. Ltd., Shanghai, China), were dissolved in deionized water to prepare metal stock solutions.

### Polymerase chain reaction (PCR)

The DNA fragment was amplified by PCR using 2 × Phanta Max Master Mix (Vazyme, Nanjing, China). The PCR mixture, with a final volume of 50 μL, contained 25 μL of 2 × Phanta Max Master Mix, 1 μL of each 10 μM primer, 50 ng of genomic DNA, and ddH_2_O. The PCR protocol included initial denaturation at 95 °C for 5 min, followed by 35 cycles of denaturation at 95 °C for 15 s, annealing at 55 °C for 15 s, and extension at 72 °C for 30 s. A final extension was performed at 72 °C for 5 min in an Applied Biosystems™ 2720 Thermal Cycler (Thermo Fisher Scientific, USA). The PCR products were electrophoresed on a 1% agarose gel stained with Goldview (Biosharp, China) Nucleic Acid Stain and scanned using a ChemiDoc XRS^+^ System (Bio-Rad, USA).

### Construction of deletion mutants and complemented strains

The gene loci of *cadX*, *cadD*, *permease*, and *FeoA* in the GZ0565 genome are *BFP66_RS01345*, *BFP66_RS01350*, *BFP66_RS01325*, and *BFP66_RS02660*, respectively. The *cadDX* deletion mutant (Δ*cadDX*) and the *permease* deletion mutant (Δ*permease*) were generated using the natural transformation method following a previous study [17]. To complement *cadDX* (C-*cadDX*), *cadD* (C-*cadD*), or *cadX* (C-*cadX*) into Δ*cadDX*, PCR fragments containing either *cadDX* or *cadD* with the *cadDX* operon promoter or *cadX* with both the *cadDX* operon promoter and its own promoter were cloned and inserted into the pSET2 plasmid [18], respectively. The recombinant plasmid was then transformed into the Δ*cadDX* strain. To construct the *FeoA* overexpression strain, a PCR fragment containing *FeoA* with its own operon promoter was cloned and inserted into the pSET2 plasmid, and the recombinant plasmid was then transformed into the GZ0565 strain (WT). The plasmids pSET2, pSET2-*cadDX*, pSET2-*cadD*, or pSET2-*cadX* were subsequently transferred into *S. agalactiae* GD201008-001, resulting in the construction of *S. agalactiae*-pSET2, *S. agalactiae*-*cadDX*, *S. agalactiae*-*cadD*, or *S. agalactiae*-*cadX*, respectively. The plasmids pSET2 or pSET2-*cadDX* were transferred into a virulence-attenuated strain of *S. agalactiae* GD201008-001 with deletion of the *CRISPR* locus (Δ*CRISPR*_*S.a*_) [19], resulting in the construction of Δ*CRISPR*_*S.a*_-pSET2 or Δ*CRISPR*_*S.a*_-*cadDX*, respectively. All primers are provided in Additional file 2.

### RNA extraction and transcriptome analysis

For transcriptome analysis, the WT and Δ*cadDX* were grown to the exponential phase (OD_600_ = 0.6) in THB. Total RNA was extracted using the FastRNA Pro Blue Kit (MP Biomedicals, USA). All the RNA samples were subsequently purified by DNase I (Takara, Dalian, China) digestion, phenol/chloroform extraction, and ethanol precipitation, as described in our previous study [20]. Two biological replicates were combined for each bacterium to create a sequencing sample, and two sequencing samples were prepared for both the WT and Δ*cadDX* groups. The RNA samples were sent to Genedenovo Technology (Guangzhou, China) for transcriptome analysis. Following the manufacturer’s protocol, sequencing was performed using the Illumina HiSeq 2500 platform (Illumina, USA). The sequencing reads were aligned by Bowtie2 [21]. Gene expression was calculated via the fragments per kilobase of transcript per million mapped reads (FPKM) algorithm by expectation–maximization (RSEM) software, and differential expression analysis was conducted using edgeR [22, 23]. Significance thresholds of* p* < 0.05 and |log_2_fold-change|≥ 1.0 were applied to identify differentially expressed genes (DEGs).

### Real-time quantitative PCR (RT-qPCR)

To evaluate the expression of *cadD* and *cadX* in *S. suis* in response to various metals, *S. suis* GZ0565 was grown to the exponential phase (OD_600_ = 0.6) in THB and then divided into seven equal aliquots, supplemented with 15 µM CdCl_2_, 0.5 mM ZnCl_2_, 1 mM CuSO_4_, 1 mM MnCl_2_, 1 mM MgCl_2_, 1 mM NiCl_2_, or deionized water, respectively. The concentrations of these heavy metals were determined on the basis of several reports on the response of *S. suis* to metals, with slight modifications [24–27]. After 1 h of incubation, the cultures were centrifuged at 5000 *g* for 10 min at 4 ℃ to collect the bacterial pellets. Total RNA isolation was performed as described above.

Approximately 0.5 μg of RNA per sample was converted to cDNA using the HiScript III 1st Strand cDNA Synthesis Kit (+ gDNA wiper) (Vazyme, Nanjing, China). Quantitative PCR was conducted using the Applied Biosystems QuantStudio 6 Flex system (Applied Biosystems, USA) with ChamQ Universal SYBR qPCR Master Mix (Vazyme, Nanjing, China). The primers used for RT-qPCR analysis are listed in Additional file 2, with the gene *BFP66_RS05620* (*parC*) serving as the internal control. The relative fold change was calculated using the 2^−ΔΔCT^ method. At least three biological replicates were included for each experiment.

### Growth curve and viability analyses

For growth curve analysis, *S. suis* was initially cultured overnight in THB. Then, they were diluted 1:100 in fresh THB or THB supplemented with different concentrations of CdCl_2_ (7.5, 15, 20, and 30 µM), ZnCl_2_ (0.25, 0.5, 1.0, and 2.0 mM), or CuSO_4_ (0.25, 0.5, 1.0, and 2.0 mM) without spectinomycin. The concentrations of these heavy metals were determined on the basis of our preliminary experiments, where high concentrations of CdCl_2_ (≥ 50 µM), ZnCl_2_ (≥ 5 mM), or CuSO_4_ (≥ 5 mM) led to complete inhibition of growth, whereas low concentrations of CdCl_2_ (≤ 5 µM), ZnCl_2_ (≤ 0.2 mM), or CuSO_4_ (≤ 0.2 mM) had no observed effect on the growth of *S. suis* strain GZ0565. For *S. agalactiae*, overnight cultures were diluted 1:100 in fresh THB or THB supplemented with 15 µM CdCl_2_ without spectinomycin. The bacterial cultures were incubated in 96-well plates at 37 °C, and the optical density at 595 nm was monitored hourly using a microplate reader (Molecular Devices, USA). Additionally, the bacterial viability of *S. suis* was assessed at 6 h through serial dilution (10^−1^ to 10^−5^) and plating onto THA plates overnight incubation. At least three biological replicates were included for each experiment.

### Expression and purification of the CadX protein

To express the CadX homodimer, a linker sequence (GGGGSGGGGSGGGGS) was inserted between two identical CadX protein sequences and then ligated into the pET28a vector to produce pET28a-CadX, following a previously established protocol [28]. *E. coli* BL21(DE3) containing the expression plasmid was cultured in 200 mL of LB supplemented with kanamycin at 37 °C until the OD_600_ reached 0.4 to 0.6. Protein expression was induced by adding 1 mM IPTG, and the culture was incubated at 16 °C for 12 h. The cells were harvested by centrifugation (5000 *g*, 10 min, 4 °C) and resuspended in 25 mL of PBS. After sonication and centrifugation (13,000 *g*, 10 min, 4 °C), the supernatant was loaded onto a HisTrap HP column (GE Healthcare, USA) and washed with buffer A (20 mM phosphate buffer, pH 7.4; 500 mM NaCl; 20 mM imidazole). Proteins were eluted with buffer E (20 mM phosphate buffer, pH 7.4; 500 mM NaCl; 500 mM imidazole). The eluted fractions were analysed by a 12% SDS gel, and the concentration of the purified protein was measured by Pierce™ BCA protein assay kits (Thermo Fisher Scientific, USA).

### Electrophoretic mobility shift assay (EMSA)

The promoter fragments of the target genes, as well as the 16S rRNA gene, were amplified by PCR with 2 × Rapid Taq Master Mix (Vazyme, Nanjing, China) from the GZ0565 genome and purified using FastPure Gel DNA Extraction Mini Kit (Vazyme, Nanjing, China). The DNA probes and CadX recombinant protein were incubated in a 20 µL reaction mixture (10 mM Tris, 50 mM KCl, 1 mM MgCl_2_, 1 mM DTT, 0.05% Triton X-100, 2.5% glycerol, pH 7.5) at 37 °C for 30 min, followed by electrophoresis on a 6% native polyacrylamide gel in 0.5 × TB buffer (44.5 mM Tris-base, 44.5 mM boric acid, pH 7.5) at 200 V for 45 min. To assess the effect of Cd^2+^ on the affinity of CadX for the *cadDX* promoter region, the DNA probes and CadX recombinant protein were incubated in the presence of 2, 4, or 8 µM CdCl_2_. The gel was stained with Goldview (Biosharp, China) for 10 min and scanned using a ChemiDoc XRS^+^ System (Bio-Rad, USA).

### β-galactosidase activity assay

The promoter fragments were amplified from the GZ0565 genome and ligated into the pTCV-*LacZ* reporter plasmid [29]. The recombinant plasmids were introduced into the WT and Δ*cadDX* strains. The β-galactosidase activity assay followed Miller’s method with certain modifications [30]. The overnight cultures were diluted 1:100 in fresh THB and incubated at 37 ℃ with 5% CO_2_. When the exponential phase was reached, the OD_600_ was measured. Subsequently, 4 mL of each culture was concentrated to 400 μL, and the culture was transferred to precooled tubes on ice. Then, 25 µL of 4 × Z-buffer (240 mM Na_2_HPO_4_·2H_2_O, 160 mM NaH_2_PO_4_·2H_2_O, 40 mM KCl, and 4 mM MgSO_4_·7H_2_O, pH 7.0) containing 50 mM β-mercaptoethanol and 4 µL of lysozyme (2.5 mg/mL) were added to the culture and incubated in a 37 ℃ water-bath for 30 min. Subsequently, 400 µL of 1 × Z-buffer containing 2 mg/mL ONPG (O-nitrophenyl-β-d-galactopyranoside) was added, and the reaction continued to incubate in a 37 ℃ water-bath for 120 min. The reaction was terminated with 400 µL of 1 M Na_2_CO_3_. After centrifugation, the supernatant was collected, and the optical density at 420 nm (OD_420_) was measured. β-galactosidase activity was calculated according to the following formula:

$$activity[MU]=\frac{O{D}_{420}\times 1000\times {V}_{E}[\mu L]}{{V}_{s}[\mu L]\times RT[\mathit{min}]\times O{D}_{600}}$$

where MU = Miller units, V_E_ = end volume, RT = reaction time, and V_S_= volume of each sample. At least three biological replicates were included for each experiment.

### 5′-RACE

To identify the transcriptional start site (TSS) of *cadX*, the SMARTer RACE 5′/3′ cDNA amplification kit (Takara, Dalian, China) was used according to the manufacturer’s instructions. Total RNA was extracted and converted to cDNA to capture the 5′ RNA ends. 5′-RACE was performed using nested PCR, which involved two rounds of PCR amplification of the cDNA. The first round of PCR was conducted with the specific primer GSP-*cadX* and the Universal Primer Short. The product was then diluted and subjected to a second round of PCR using NGSP-*cadX* and the Universal Primer Short. The PCR products were separated on 1.5% agarose gels and purified by a FastPure Gel DNA Extraction Mini Kit (Vazyme, Nanjing, China). The purified fragments were ligated into the linearized pMD19T vector (Takara, Dalian, China). After sequencing (Sangon Biotech, Shanghai, China) and alignment to the GZ0565 genome, the 5′ end of *cadX* was identified. The primers used are listed in Additional file 2.

### Oxidative stress assay

To evaluate the role of *cadDX* in the oxidative stress response, bacteria were challenged with H_2_O_2_. All the strains were cultured to the exponential phase (OD_600_ = 0.6). H_2_O_2_ was subsequently added to achieve a final concentration of 25 mM in THB. After incubation at 37 ℃ for 25 min, bacterial counts were determined by spreading serial dilutions on THA plates. The survival rate at each time point was calculated as CFU at 25 min/CFU at time point 0. At least three biological replicates were included for each experiment.

### Protein sequence alignment and phylogenetic analysis

The CadX and CadD protein sequences were aligned using BLASTp. The selected homologous sequences were further aligned using Clustal Omega [31], and a maximum-likelihood phylogenetic tree was constructed using MEGA X software [32]. The serotypes of 191 *S. suis* strains, which were randomly chosen from the BLASTp results, were analysed on the basis of their variation in capsular polysaccharide (CPS) antigenicity [33]. Information on *S. suis* strains with *cadDX* used for serotype distributions and bacteria with *cadDX* used for phylogenetic analysis is provided in Additional file 3 and Additional file 4, respectively.

### Determination of viable bacteria in organs

The virulence of *S. suis* (WT, Δ*cadDX*, and C-*cadDX*) and *S. agalactiae* (Δ*CRISPR*_*S.a*_*-*pSET2 and Δ*CRISPR*_*S.a*_*-cadDX*) was assessed in mice. Mouse infection was carried out at the Laboratory Animal Center of Nanjing Agricultural University with the approval of the institution's ethics committee (permit number SYXK (Su) 2021–0086). Bacteria were cultured to the exponential phase (OD_600_ = 0.6), washed twice with PBS, and used for infection in six-week-old SPF CD1 female mice (SiPeiFu Biotechnology, Shanghai, China), with six mice per group for *S. suis* and five mice per group for *S. agalactiae*. For *S. suis*, the mice were intraperitoneally injected with 1.5 × 10^8^ CFU of *S. suis* following a previously established protocol [34]. For *S. agalactiae*, the mice were intraperitoneally injected with 1.0 × 10^2^ CFU of *S. agalactiae* due to its high virulence in the mouse infection model, as reported in a published study [19]. All the mice were euthanized at 24 h post-infection. Blood samples (20 μL) were collected from the heart; liver, kidney, and brain samples were taken, weighed, suspended in PBS, and homogenized. The number of viable bacteria in organs and blood was determined by plating serial dilutions onto THA.

### Statistical analyses

For experiments with three groups or more than three groups, comparisons between groups were conducted via one-way ANOVA followed by Dunnett’s multiple comparisons test. For experiments with only two groups, such as the β-galactosidase activity assay and determination of *S. agalactiae* in organs, unpaired two-tailed Student’s *t* tests were used. Before performing unpaired two-tailed Student’s *t* tests, we tested for equal variances via an F test. Statistical analyses were performed using GraphPad Prism version 8 software. The data are presented as mean ± SD, and statistical significance was set at *p* < 0.05.

## Results

### Expression of *cadD* and *cadX* in *S. suis* under different metal conditions

**Figure 1:**
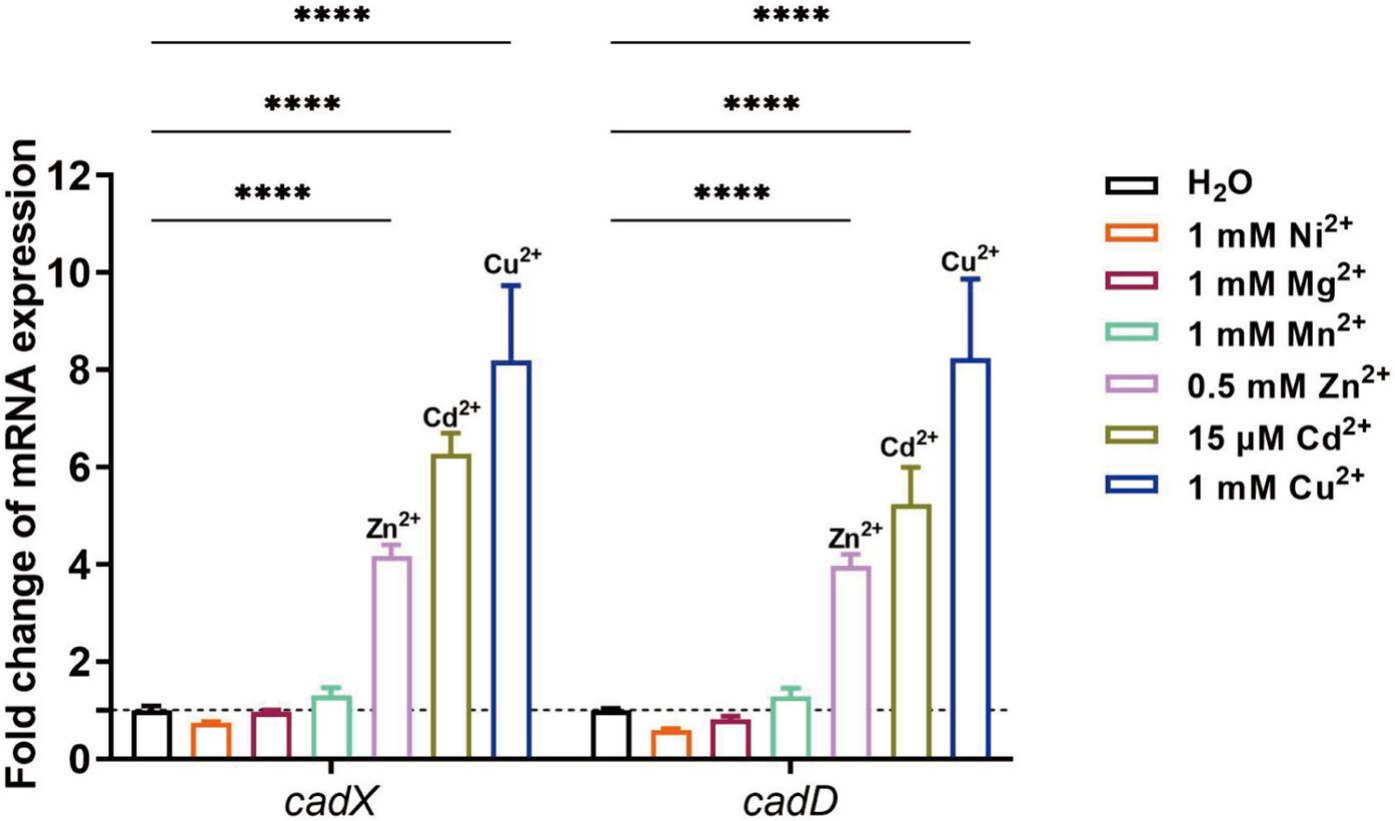
**Expression of *cadD* and *cadX* in *S. suis* under different metal conditions.** The expression of *cadD* and *cadX* in the WT strain was determined under H_2_O (control) or different metal conditions (15 µM CdCl_2_, 0.5 mM ZnCl_2_, 1 mM CuSO_4_, 1 mM MnCl_2_, 1 mM MgCl_2_, and 1 mM NiCl_2_). Data are presented as mean ± SD, and asterisks indicate significantly different values (“****” indicates *p* < 0.0001).

To explore the involvement of *cadDX* in the response of *S. suis* to different metals, RT-qPCR was used to analyse the expression of *cadD* and *cadX* in the WT strain treated with various metals. The expression of *cadD* in *S. suis* was upregulated 5.23-fold in response to 15 µM CdCl_2_, 3.97-fold in response to 0.5 mM ZnCl_2_, and 8.35-fold in response to 1 mM CuSO_4_ (Figure 1). Similarly, the expression of *cadX* in *S. suis* was upregulated 6.27-fold in response to 15 µM CdCl_2_, 4.17-fold in response to 0.5 mM ZnCl_2_, and 8.20-fold in response to 1 mM CuSO_4_ (Figure 1). In contrast, the expression of *cadD* and *cadX* was not induced by 1 mM MnCl_2_, 1 mM MgCl_2_, or 1 mM NiCl_2_ (Figure 1). These results suggest that *cadDX* may be involved in the response of *S. suis* to stress induced by Cd^2+^, Zn^2+^, or Cu^2+^.

### *cadDX* protects *S. suis* against cadmium stress

**Figure 2:**
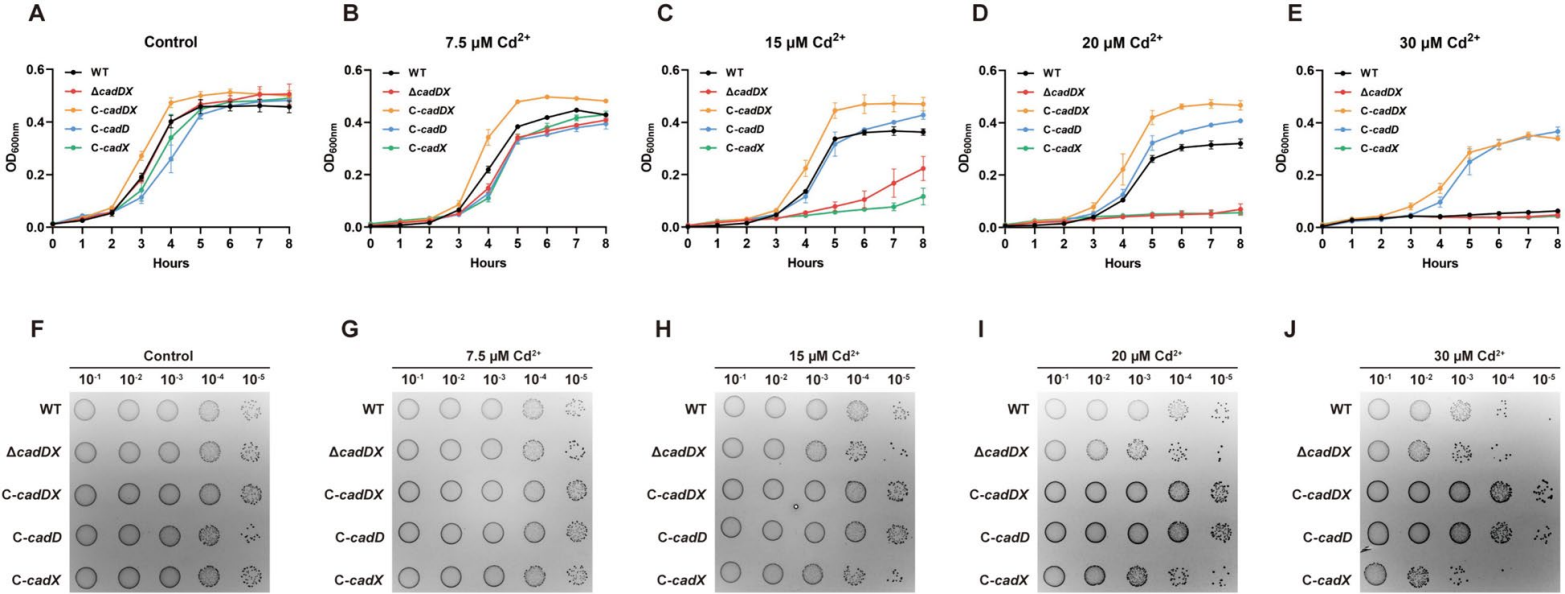
***cadDX* protects *S. suis* against cadmium stress. A**–**E** Growth curves of WT, Δ*cadDX*, C-*cadDX*, C-*cadD*, and C-*cadX* in THB (Control) or THB supplemented with various concentrations of CdCl_2_ (7.5, 15, 20, and 30 µM). **F**–**J** Bacterial viability of the WT, Δ*cadDX*, C-*cadDX*, C-*cadD*, and C-*cadX* strains after 6 h of culture in THB (control) or THB supplemented with various concentrations of CdCl_2_ (7.5, 15, 20, and 30 µM).

The growth of the WT, Δ*cadDX*, C-*cadDX*, C-*cadD*, and C-*cadX* strains in THB media supplemented with different concentrations of CdCl_2_ (7.5, 15, 20, or 30 µM), ZnCl_2_ (0.25, 0.5, 1.0, or 2.0 mM), or CuSO_4_ (0.25, 0.5, 1.0, or 2.0 mM) was evaluated to further investigate the role of *cadDX* in the *S. suis* response to excessive metals. All the strains exhibited similar growth in THB (Figure 2A). When 7.5 µM CdCl_2_ was added to the THB, the C-*cadDX* strain grew better than the Δ*cadDX*, C-*cadD*, and C-*cadX* strains did (Figure 2B). With the addition of 15 or 20 µM CdCl_2_, the WT, C-*cadD*, and C-*cadDX* strains demonstrated superior growth compared with the Δ*cadDX* and C-*cadX* strains (Figures 2C, D). At 30 µM CdCl_2_, the C-*cadD* and C-*cadDX* strains exhibited better growth than the WT, Δ*cadDX*, and C-*cadX* strains did (Figure 2E). Bacterial viability was determined to further evaluate the role of *cadDX* in resistance to Cd^2+^ (Figures 2F–J). Following a 6-h treatment with 15, 20, or 30 µM CdCl_2_, the Δ*cadDX* and C-*cadX* strains formed fewer colonies than the WT, C-*cadD*, and C-*cadDX* strains did. However, all strains exhibited similar growth curves under ZnCl_2_ or CuSO_4_ conditions (0.25, 0.5, 1.0, or 2.0 mM), indicating that *cadDX* does not play a pivotal role in the response to Zn^2+^ or Cu^2+^ stress in *S. suis* (Additional files 5A–H).

### Autoregulatory mechanism of *cadDX* in cadmium resistance

**Figure 3:**
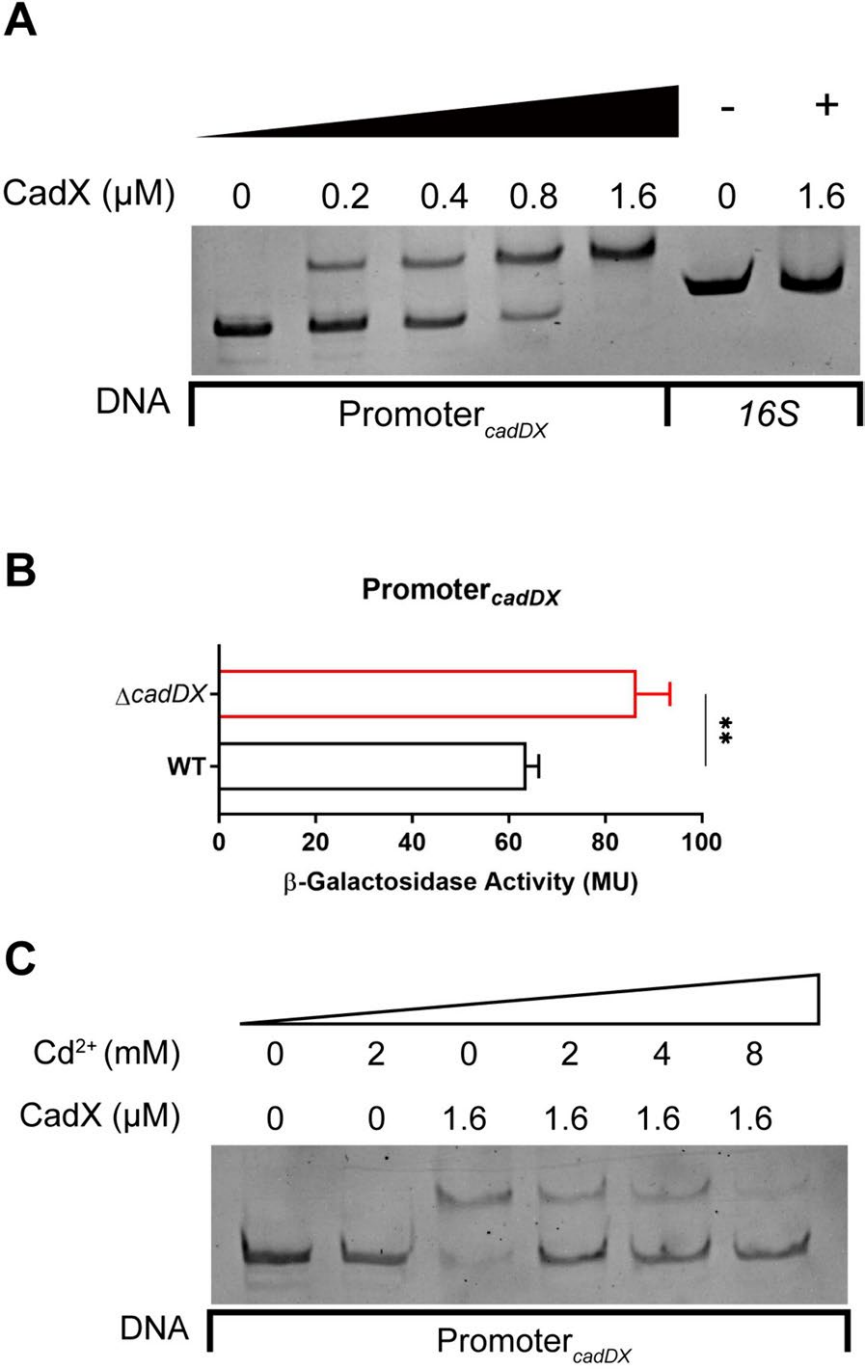
**Autoregulatory mechanism of *cadDX* in cadmium resistance. A** CadX directly binds to the *cadDX* promoter. **B** The activity of β-galactosidase under the control of the *cadDX* promoter in the WT and Δ*cadDX* strains. **C** Cd^2+^ reduces the affinity of CadX for the *cadDX* promoter. Data are presented as mean ± SD, and asterisks indicate significantly different values (“**” indicates *p* < 0.01).

To elucidate the regulatory mechanism of *cadDX* in cadmium resistance, we initially expressed, purified the CadX protein, and amplified a DNA fragment containing the *cadDX* promoter. We then assessed the interaction between the CadX protein and the *cadDX* promoter region by EMSA. The EMSA results revealed that CadX directly binds to the *cadDX* promoter region (Figure 3A). Moreover, the activity of β-galactosidase under the control of the *cadDX* promoter was significantly greater in the Δ*cadDX* strain than in the WT strain (Figure 3B). Additionally, a previous study reported that the affinity of CadX for the *cadDX* promoter region was influenced by the presence of Cd^2+^ [12]. Consistent with these findings, the EMSA results indicated that the affinity of CadX for the *cadDX* promoter region was significantly reduced in the presence of Cd^2+^ (Figure 3C).

### *cadDX* contributes to oxidative stress resistance

**Figure 4:**
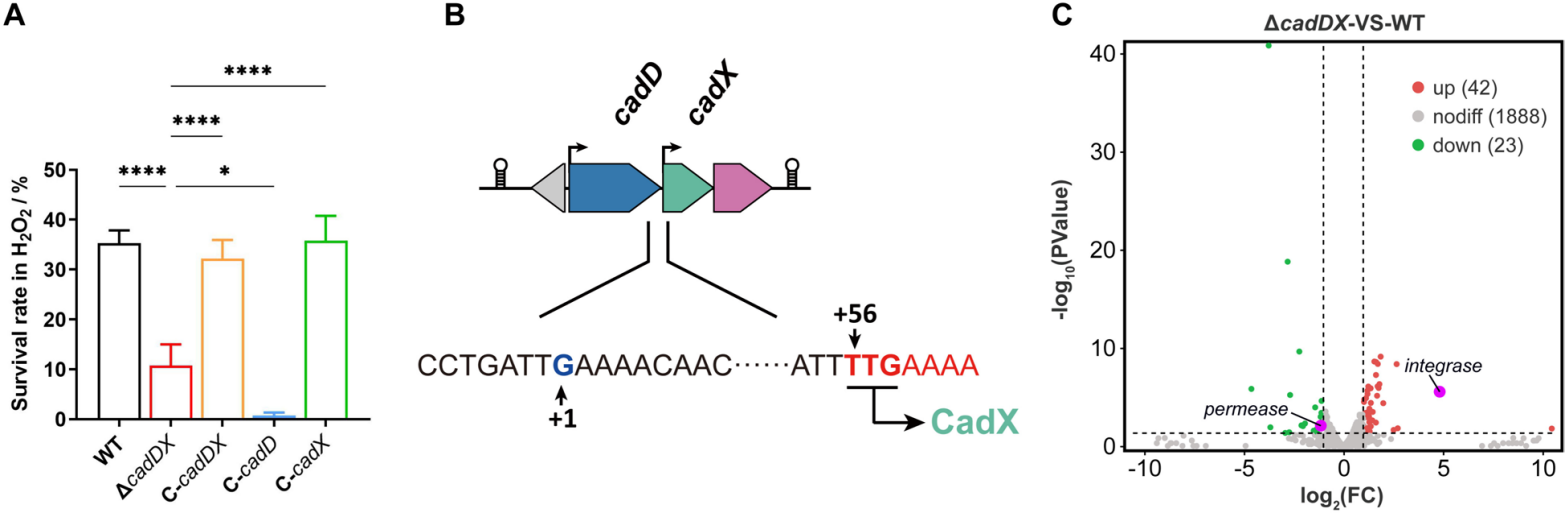
***cadDX* contributes to oxidative stress resistance. A** Survival rates of the WT, Δ*cadDX*, C-*cadDX*, C-*cadD*, and C-*cadX* strains under H_2_O_2_ stress conditions. **B** An additional promoter of *cadX* inside the *cadDX* operon. “TTG” is the start codon of CadX. “ + 1” indicates the position of the identified additional TSS. **C** Volcano plot demonstrating DEGs in the Δ*cadDX* strain compared with the WT strain by transcriptome analysis. The cut-off for enrichment was set at |Log_2_(FC)|≥ 1.0 and a *p* value < 0.05, as indicated by the dashed lines. The upregulated genes are marked with red dots, the downregulated genes are marked with green dots, and the DEGs within the 11K IME are marked with purple dots. Data are presented as mean ± SD, and asterisks indicate significantly different values (“*” and “****” indicate *p* < 0.05 and *p* < 0.0001, respectively).

In our recent study, *cadD* (2.4-fold change) and *cadX* (3.4-fold change) were found to be upregulated in response to H_2_O_2_ stress compared with those in THB medium by transcriptome analysis, suggesting potential functions of *cadDX* under oxidative stress [35]. Thus, we evaluated the survival of the WT, Δ*cadDX*, C-*cadDX*, C-*cadD*, and C-*cadX* strains under H_2_O_2_ conditions. The results revealed that, compared with the WT and *C-cadDX* strains, the Δ*cadDX* strain presented a significantly lower survival rate after H_2_O_2_ treatment (Figure 4A). Additionally, *cadX* complementation alone in Δ*cadDX* restored its resistance to H_2_O_2_ to the WT level, while C-*cadD* further reduced resistance to H_2_O_2_ (Figure 4A). Compared with that of C-*cadDX*, *cadD* expression was substantially increased in C-*cadD* because of the absence of *cadX* (Additional file 6A). Given the divergent expression patterns of *cadD* and *cadX* during oxidative stress (2.4-fold and 3.4-fold changes, respectively), we hypothesized that *cadX* might have an additional promoter within the *cadDX* operon. To explore this hypothesis, we conducted a 5′ RACE analysis to pinpoint the TSS of *cadX* (Additional file 6B). We identified an additional TSS at -56 relative to the *cadX* start codon (Figure 4B and Additional file 6C). However, we observed no interaction between CadX and its own promoter (Additional file 6D). The activity of β-galactosidase under the control of the *cadX* promoter in the Δ*cadDX* strain was comparable to that in the WT strain (Additional file 6E).

To explore the influence of the *cadDX* operon on gene expression in *S. suis*, we conducted transcriptome analysis of the WT and Δ*cadDX *strain. In the absence of *cadDX*, we identified 65 DEGs, consisting of 42 upregulated and 23 downregulated genes (Figure 4C) (Additional file 7). To confirm the reliability of the transcriptome data, seven upregulated and six downregulated DEGs were randomly selected for further analysis. The transcription levels of these genes were consistent with those obtained from the transcriptome analysis (Additional file 8), indicating the reliability of the transcriptome data. The 65 DEGs are involved in various biological processes, including fatty acid biosynthesis, carbohydrate transport and metabolism, and metal ion transport. Notably, among these DEGs, only *permease* (encoded by *BFP66_RS01325*) and *integrase* (encoded by *BFP66_RS01340*) were located within the 11 K IME (Figure 4C), which suggests that *cadDX* mainly affects the expression of bacterial genomic genes outside the 11K IME. As shown in Additional file 7, six genes related to the PTS transporter system (encoded by *BFP66_RS02205*, *BFP66_RS02210*, *BFP66_RS02215*, *BFP66_RS02220*, *BFP66_RS02225*, and *BFP66_RS02230*) were upregulated in Δ*cadDX*. The genes associated with fatty acid biosynthesis (encoded by *BFP66_RS08345*, *BFP66_RS08350*, and *BFP66_RS08355*) were downregulated. We also found that ferrous iron transport *FeoA* (encoded by *BFP66_RS02660*) was upregulated and that *permease* was downregulated in Δ*cadDX*. Furthermore, the activity of β-galactosidase under the control of the promoter of *Fab* (*BFP66_RS08355*, the first gene of the operon involved in fatty acid biosynthesis) or *permease* was decreased in Δ*cadDX* (Additional files 9A and B). In contrast, the activity of β-galactosidase under the control of the promoter of *PTS* (*BFP66_RS02205*, the first gene of the operon involved in the PTS transporter system) or *FeoA* was increased (Additional files 9C and D). However, CadX did not directly bind to the promoters of these genes (Additional files 9E-H). Furthermore, we selected *permease* and *FeoA* for further analysis on the basis of their functions, which may be involved in oxidative stress resistance. After the *permease* deletion strain (Δ*permease*) and the *FeoA* overexpression strain (OE-*FeoA*) were subjected to H_2_O_2_ treatment, both strains presented significantly lower survival rates than the WT strain did (Additional file 10).

### *cadDX* contributes to *S. suis* virulence in a mouse infection model

**Figure 5:**
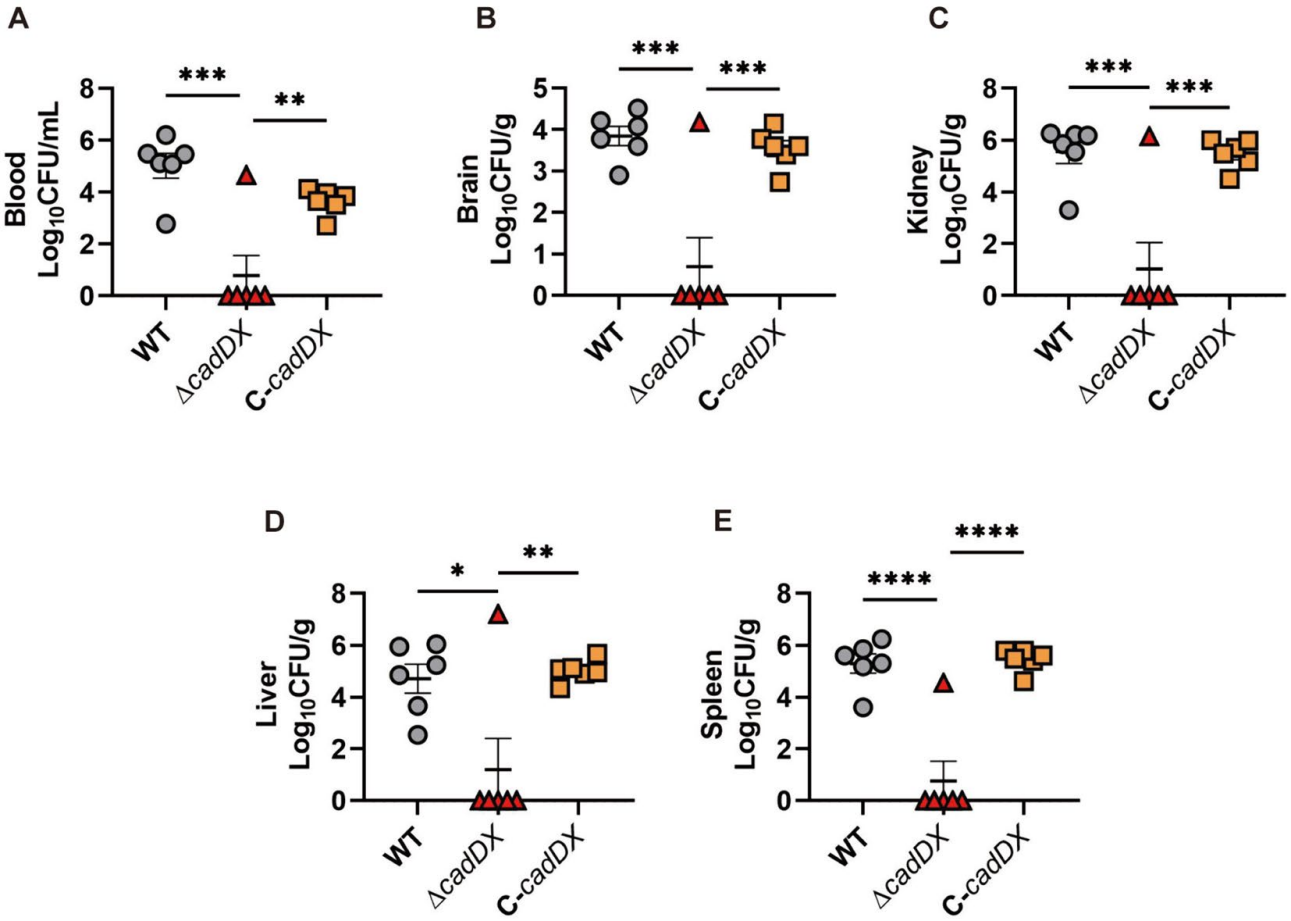
***cadDX* contributes to *S. suis* virulence in a mouse infection model.** Six mice per group were injected intraperitoneally with 1.5 × 10^8^ CFU of the WT, Δ*cadDX*, or C-*cadDX* strains. All the mice were euthanized at 24 h post-infection. Bacteria from the blood (**A**), brain (**B**), kidney (**C**), liver (**D**), and spleen (**E**) were plated onto THA, and colonies are expressed as Log_10_CFU/g or Log_10_CFU/mL. Data are presented as mean ± SD, and asterisks indicate significantly different values (“*”, “**”, “***”, and “****” indicate *p* < 0.05, *p* < 0.01, *p* < 0.001, and *p* < 0.0001, respectively).

Given the role of *cadDX* in alleviating oxidative stress in *S. suis*, we investigated its contribution to *S. suis* virulence in a mouse model. Compared with those in the WT and C-*cadDX* infection groups, the number of Δ*cadDX* bacteria in the blood, brain, kidney, liver, and spleen was significantly lower (Figures 5A–E).

### Characterization of the 11K IME in *S. suis* GZ0565

**Figure 6:**
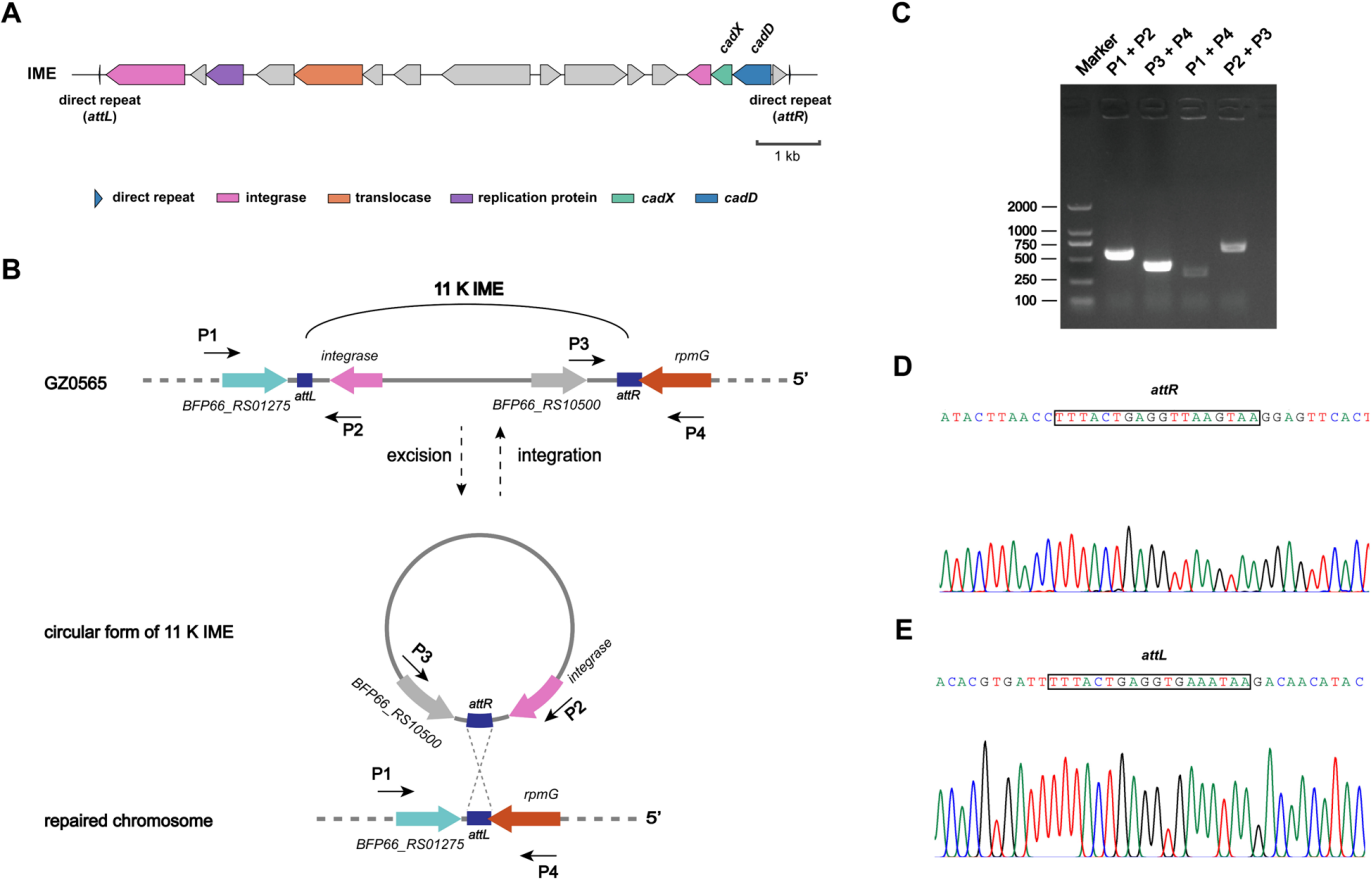
**Characterization of the 11K IME in *S. suis* GZ0565.** **A** The 11K IME contains direct repeats, a site-specific integrase, a replication protein, and a translocase. **B** Illustration of the site-specific integration and excision of the 11K IME. The locations and orientations of the primers used for detecting integrated and excised 11K are indicated by arrows. **C** Detection of a circular extrachromosomal form of the 11K IME by PCR analysis. **D** Sequencing chromatogram of PCR products amplified with the primer pair P2/P3 showing the *attR* site (boxed). **E** Sequencing chromatogram of PCR products amplified with the primer pair P1/P4 showing the *attL* site (box) upon 11K IME excision.

In the *S. suis* virulent strain GZ0565, we identified the *cadDX* operon within an 11 K IME that contains characteristics of IME, including site-specific integrases, replication proteins, and translocases (Figure 6A). Direct repeats (*attL* and *attR*) were detected on both sides of this IME, suggesting the potential for circularization of the 11 K IME and subsequent transfer to new hosts. We designed two pairs of primers to assess their circularization ability. We confirmed that the 11 K IME could excise from the bacterial chromosome, forming an extrachromosomal circular molecule (Figures 6B, C). Subsequent sequence analysis validated the expected structures of *attR* (Figure 6D) and *attL* (Figure 6E) generated during site-specific excision.

### Distribution of *cadDX* in bacteria

**Figure 7:**
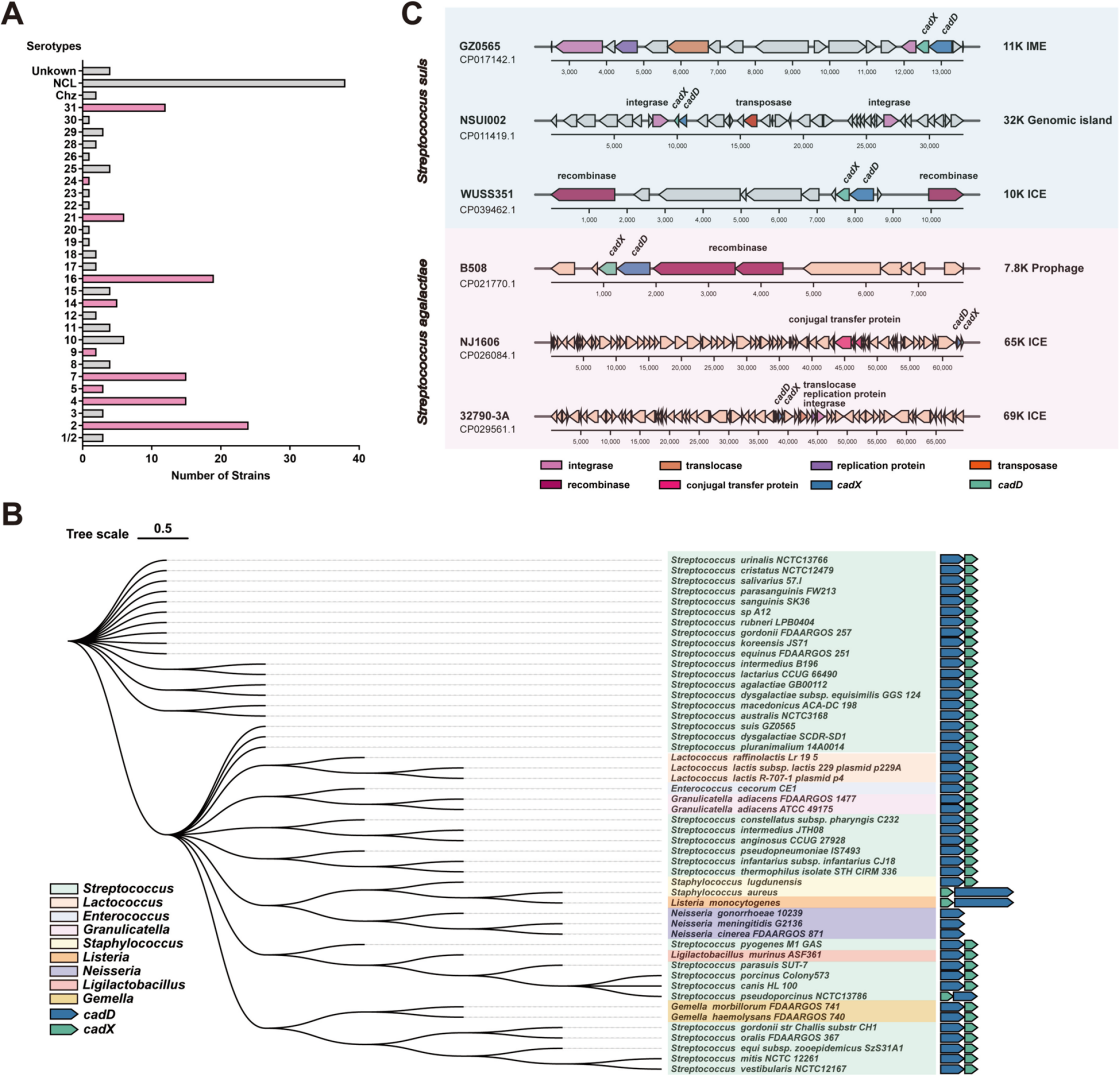
**Distribution of *cadDX*** **in bacteria. A** The presence of *cadDX* in different *S. suis* serotypes. **B** The presence of *cadDX* in different gram-positive bacteria, including *Streptococcus*, *Lactococcus*, *Enterococcus*, *Staphylococcus*, *Listeria*, *Ligilactobacillus*, and *Gemella*, and the gram-negative bacterium *Neisseria*. **C** *cadDX* within different vectors in *S. suis* and *S. agalactiae*.

Genome analysis revealed the prevalence of the *cadDX* operon in diverse *S. suis* serotypes, including ten serotypes (serotypes 2, 4, 5, 7, 9, 14, 16, 21, 24, and 31) known to cause human infections (Figure 7A). Except for the gram-negative bacteria *Neisseria,* where *cadD* is an orphan gene, the *cadDX* operon is present in various gram-positive bacteria, including *Streptococcus*, *Lactococcus*, *Enterococcus*, *Staphylococcus*, *Listeria*, *Ligilactobacillus*, and *Gemella*. Among these gram-positive bacteria, pathogenic streptococci such as *Streptococcus pyogenes*, *Streptococcus dysgalactiae*, *Streptococcus equi* subsp. *zooepidemicus*, and *S. agalactiae* present the highest prevalence of the *cadDX* operon (Figure 7B). The widespread distribution of *cadDX* indicates horizontal transfer across diverse bacteria. Importantly, this horizontal transfer of *cadDX* can occur through various vectors. In *S. suis* strain WUSS351, *cadDX* resides within a predicted 10 K ICE containing recombinase, which differs from its location in the *S. suis* strain GZ0565. Similarly, another 32 K genomic island with an integrase and transposase also contains *cadDX* in the *S. suis* strain NSUI002. A similar trend was observed in *S. agalactiae* strains NJ1606 and 32790-3A, where *cadDX* was identified in two different ICEs along with several genes associated with horizontal transfer, such as conjugal transfer proteins, translocases, replication proteins, and integrases. Additionally, *cadDX* was found within a prophage containing recombinase in *S. agalactiae* strain B508 (Figure 7C). Overall, these findings underscore the significant variability in the distribution and genetic context of *cadDX* among gram-positive bacteria, especially pathogenic streptococci. This diversity has substantial implications for the evolution and functional roles of *cadDX* across distinct bacterial populations.

### *cadDX* confers resistance to cadmium and oxidative stresses and enhances virulence in *S. agalactiae*

**Figure 8:**
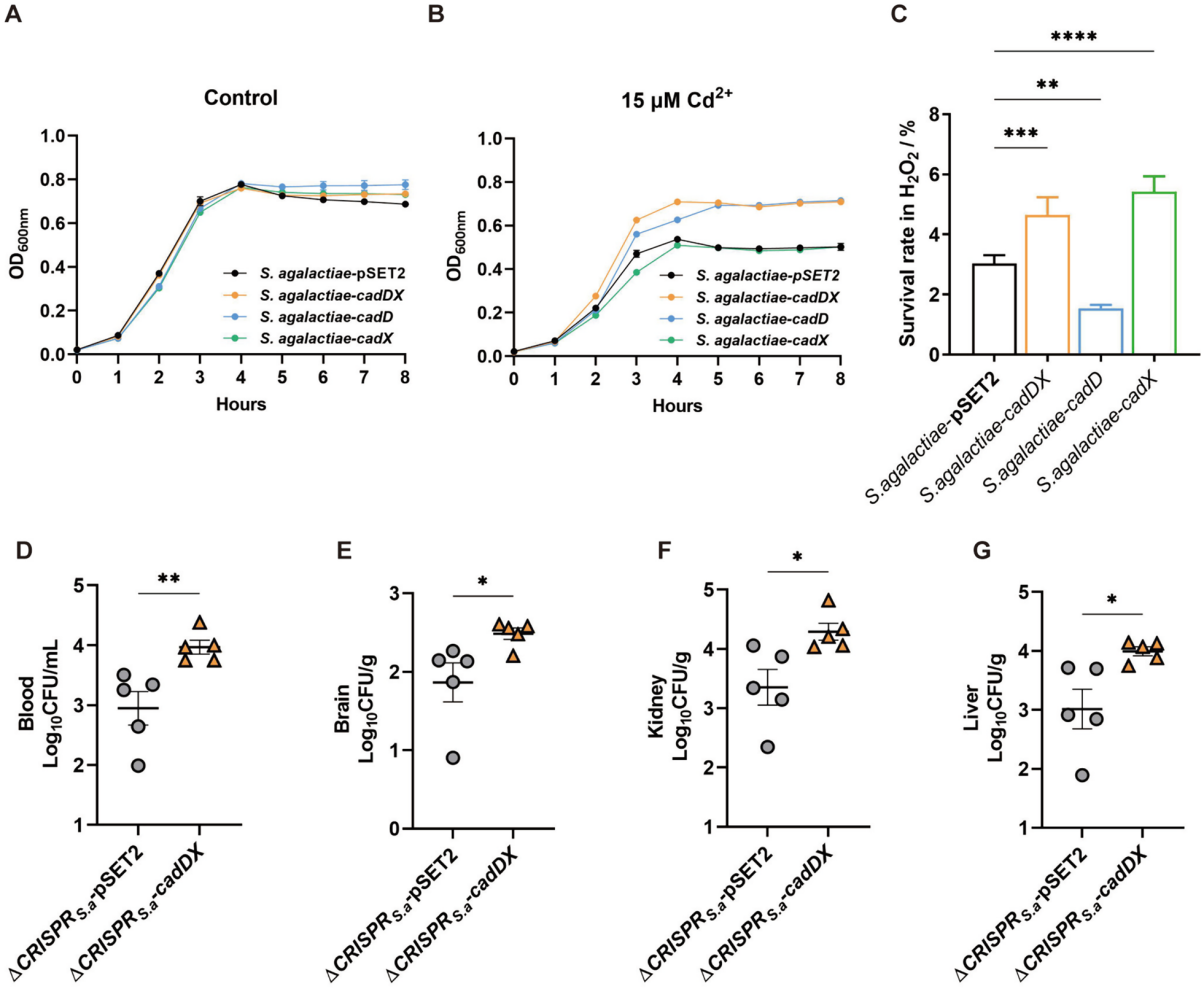
***cadDX*** **confers resistance to cadmium and oxidative stress and enhances virulence in *S. agalactiae***. **A**, **B** Growth curves of *S. agalactiae*-pSET2, *S. agalactiae*-*cadDX*, *S. agalactiae*-*cadD*, and *S. agalactiae*-*cadX* cultured in THB (control) or THB supplemented with 15 µM CdCl_2_. **C** Survival rates of *S. agalactiae*-pSET2, *S. agalactiae*-*cadDX*, *S. agalactiae*-*cadD*, and *S. agalactiae*-*cadX* under H_2_O_2_ stress conditions. Five mice per group were injected intraperitoneally with 1.0 × 10^2^ CFU of Δ*CRISPR*_*S.a*_*-*pSET2 or Δ*CRISPR*_*S.a*_*-cadDX*. All the mice were euthanized at 24 h post-infection. Bacteria from the blood (**D**), brain (**E**), kidney (**F**), and liver (**G**) were plated onto THA, and colonies are expressed as Log_10_ CFU/g or Log_10_ CFU/mL. Data are presented as mean ± SD, and asterisks indicate significantly different values (“*”, “**”, “***” and “****” indicate *p* < 0.05, *p* < 0.01, *p* < 0.001, and *p* < 0.0001, respectively).

To investigate whether *cadDX* could confer similar functions in recipient bacteria lacking this operon, we introduced *cadDX* into *the S. agalactiae* strain GD201008-001 using the pSET2 plasmid. In THB media, *S. agalactiae*-pSET2, *S. agalactiae*-*cadDX*, *S. agalactiae*-*cadD*, and *S. agalactiae*-*cadX* displayed comparable growth curves (Figure 8A). However, when cultured in THB containing 15 µM CdCl_2_, the growth of *S. agalactiae*-*cadD* and *S. agalactiae*-*cadDX* were notably greater than those of *S. agalactiae-*pSET2 and *S. agalactiae*-*cadX* (Figure 8B). Similarly, the survival rates of *S. agalactiae-cadX* and *S. agalactiae*-*cadDX* increased under H_2_O_2_ conditions compared with those of *S. agalactiae*-pSET2 (Figure 8C). Moreover, compared with *S. agalactiae*-pSET2, *S. agalactiae*-*cadD* also exhibited increased sensitivity to H_2_O_2_. Notably, owing to the high virulence of the *S. agalactiae* strain GD201008-001 in a mouse infection model, with a 50% lethal dose value of less than 10 CFU, we employed the virulence-attenuated strain Δ*CRISPR*_*S.a*_ as a background to assess the contributions of *cadDX* to *S. agalactiae* virulence [19, 36]. Compared with those in the Δ*CRISPR*_*S.a*_-pSET2 infection group, the number of bacteria in the blood, brain, kidney, and liver in the Δ*CRISPR*_*S.a*_*-cadDX* infection group was significantly greater (Figures 8D–G), although no significant difference was observed in the spleen (Additional file 11). These results demonstrate that *cadDX* also confers resistance to cadmium and oxidative stresses and enhances virulence in *S. agalactiae*.

## Discussion

Cadmium, a prevalent environmental pollutant, is found worldwide, particularly in areas near water resources [37]. It is a frequent contaminant in animal feed additives, often appearing as an impurity in mineral supplements such as phosphates, zinc sulfate, and zinc oxide; these supplements are commonly used in modern swine farming practices [38]. It exerts toxicity by binding to sulfhydryl groups on essential proteins, inhibiting respiratory processes [39]. Moreover, cadmium also induces oxidative damage and weakens the survival capacity of microbes [40].

Among gram-positive bacteria, two common cadmium resistance systems are *cadDX* (also called *cadBX*) and *cadCA*, both of which are typically carried on plasmids. Within the plasmid pI258 of *S. aureus*, the *cadCA* system comprises a repressor, CadC, and an efflux protein, CadA. *S. aureus* CadC shares 35% amino acid identity with *S. suis* CadX. The cadC gene encoded by the pI258 plasmid has been shown to bind metal ions, including Cd^2+^, Zn^2+^, lead (Pb^2+^), and bismuth (Bi^3+^), leading to derepression of the *cadCA* system [41, 42]. The *cadCA* system confers resistance to Cd^2+^, Zn^2+^, Pb^2+^, and Bi^3+^ in *S. aureus* [11, 43]. Unlike *S. aureus*, the *Listeria monocytogenes* plasmid pLm74 has a homologous *cadCA* system that specifically confers cadmium resistance [44]. The other system, *cadDX,* consists of the efflux protein CadD and the regulator protein CadX. In *S. lugdunensis*, the pLUG10 plasmid encodes CadX and CadD, which share 46% and 56% amino acid identity with *S. suis* CadX and CadD, respectively [7]. The *cadDX* system in *S. lugdunensis* effectively contributes to resistance against Cd^2+^ [7]. In *S. salivarius*, CadDX, which shares 98% amino acid identity with that in *S. suis*, confers resistance to both Cd^2+^ and Zn^2+^ [12]. A recent study on *S. agalactiae* also demonstrated that CadD, which shares 98% amino acid identity with that in *S. suis*, contributes to tolerance to metal toxicity, including Zn^2+^, Cd^2+^, Cu^2+^, Co^2+^, and Ni^2+^ [13]. In this study, no observable effect of *cadDX* on zinc resistance was noted in *S. suis*. *S. suis* might rely on other compensatory resistance mechanisms to combat Zn^2+^. Indeed, multiple zinc resistance mechanisms have been reported in *S. suis*. For example, Zheng et al. reported that TroR negatively regulates the *TroABCD* system, which is crucial for resistance to Zn^2+^ toxicity [25]. Another study demonstrated that AdcR negatively regulates the expression of *adcA* and *adcAII*, contributing to Zn^2+^ acquisition and virulence [45]. Furthermore, the Zn^2+^-response regulator Zur plays a role in precise Zn^2+^ homeostasis [46]. PmtA, which potentially affects the expression of the *Zur* regulon, is also involved in Zn^2+^ transport [24].

In addition to its role in conferring cadmium resistance, *cadDX* also plays a pivotal but previously unreported role in oxidative stress resistance. Oxidative stress, a common challenge faced by *S. suis* during infection, can damage cellular components by oxidizing amino acids, DNA, and lipids. In this study, we discovered that the *cadDX* operon, situated within the 11K IME, governs core genomic genes involved in resisting oxidative stress. The *FeoAB* system, a major ferrous iron transport system in pathogenic bacteria, is critical for intracellular survival and virulence [47, 48]. However, excessive intracellular ferrous iron can lead to the formation of hydroxyl radicals by reducing H_2_O_2_ in the Fenton reaction [49]. Hydroxyl radicals are highly potent oxidants of cellular macromolecules [50]. Studies on *Porphyromonas gingivalis* and *Riemerella anatipestifer* have confirmed that knockout of *FeoAB* increases bacteria resistance to H_2_O_2_ stress [51, 52]. Our previous data also showed that *FeoAB* was downregulated in response to oxidative stress [35]. Therefore, it is possible that *cadDX* represses the expression of *FeoA* to protect *S. suis* against oxidative stress resulting from excessive intracellular ferrous iron. Furthermore, *cadDX* stimulates the expression of genes involved in fatty acid synthesis. Fatty acids are essential constituents of bacterial membranes [53, 54]. By modulating their membrane composition, bacteria can effectively respond to various environmental stresses, such as oxidative stress [55]. Our previous study demonstrated that *S. suis* curtails energy-consuming pathways to conserve energy for H_2_O_2_ detoxification [35]. In this study, *cadDX* might also inhibit energy-consuming pathways (six genes related to the PTS) to conserve energy for vital metabolic processes in the context of oxidative stress. Additionally, the permease of *Nitratiruptor* sp. SB155‐2, which shares 41% amino acid identity with that of *S. suis*, was upregulated under cadmium or copper stress conditions [56], suggesting its potential role in maintaining metal homeostasis. Metal homeostasis is important for redox balance, so we speculated that *permeases* may also be involved in oxidative stress resistance. However, further investigations are needed to elucidate how the permease contributes to resistance against oxidative stress. The expression of *cadD* in the C-*cadD* strain was approximately 30-fold greater than that in the C-*cadDX* strain (Additional file 6A). The excessive expression of *cadD* may consume more ATP as an efflux pump, which is disadvantageous for combating oxidative stress. Additionally, excessive expression of *cadD* may disrupt metal homeostasis, leading to reduced resistance to H_2_O_2_ [57]. Thus, the repression of *cadD* by CadX is vital for combating oxidative stress. Notably, we determined that *cadX* harbors its own promoter and assists in combating oxidative stress by preventing the excessive expression of *cadD*. Furthermore, transcriptome analysis indicated that CadX may influence several genes involved in oxidative stress resistance, such as *permease*, *FeoA*, fatty acid synthesis-related genes, and PTS transport system-related genes, thereby contributing to oxidative stress resistance. However, further investigations are needed to elucidate the mechanisms by which *cadX* contributes to oxidative stress resistance.

Our investigations revealed that *cadDX* is located within various MGEs, including IMEs, prophages, genomic islands, ICEs, and plasmids. This diversity of vectors implies that the *cadDX* operon has important implications for evolution and function within different bacterial populations, underscoring its potential significance for bacterial adaptation and survival strategies.

**Figure 9:**
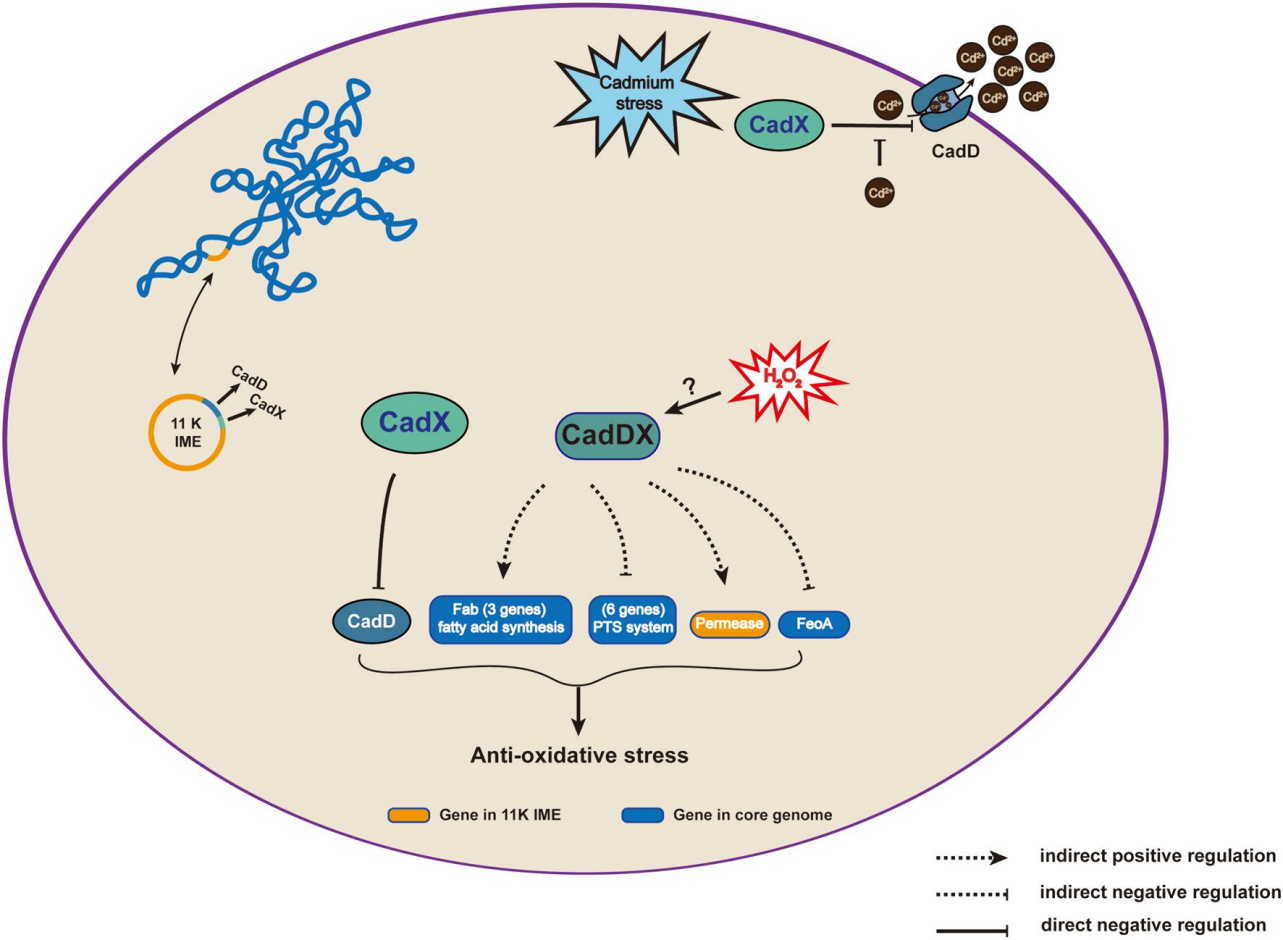
**Functions and regulatory mechanisms of *cadDX.*** In *S. suis*, *cadDX* is located within an 11K MGE that can autonomously form a circular structure. The presence of Cd^2+^ disrupts the repression of *cadD* by CadX, thereby contributing to cadmium resistance. CadX protects *S. suis* against oxidative stress by repressing *cadD* to prevent its excessive expression, which can be detrimental to the bacterium. Additionally, *cadDX* influences genes involved in the oxidative stress response, including fatty acid synthesis-related genes, PTS transport system-related genes, *permeases*, and *FeoA*, which contribute to oxidative stress resistance.

In summary, as shown in Figure 9, in addition to investigating cadmium detoxification, we revealed new functions and regulatory mechanisms of the *cadDX* operon in oxidative stress resistance and virulence in *S. suis* and *S. agalactiae*. Furthermore, we identified the *cadDX* operon in diverse MGEs, accounting for its widespread distribution across various bacteria. These findings underscore the importance of the *cadDX* operon in shaping bacterial adaptation and survival strategies.

## Supplementary Information

**Additional file 1. Bacterial strains and plasmids used in this study.**

**Additional file 2. Primers used in this study.**

**Additional file 3. Information on S. suis strains with cadDX.**

**Additional file 4. Information on bacteria with cadDX.**

**Additional file 5. WT, ΔcadDX, C-cadDX, C-cadD, and C-cadX respond to zinc and copper stress.** (A-D) Growth curves of the WT, Δ*cadDX*, C-*cadDX*, C-*cadD*, and C-*cadX* strains in THB supplemented with various concentrations of ZnCl_2_ (0.25, 0.5, 1.0, and 2.0 mM). (E-H) Growth curves of the WT, Δ*cadDX*, C-*cadDX*, C-*cadD*, and C-*cadX* strains in THB supplemented with various concentrations of CuSO_4_ (0.25, 0.5, 1.0, and 2.0 mM).

**Additional file 6. cadX possesses its own promoter. **(A) Expression of *cadD* in C-*cadDX* and C-*cadD*. Data are presented as mean ± SD, and asterisks indicate significantly different values (“**” indicates *p* < 0.01). (B) M: Molecular weight markers. Lines 1 and 3: 5′-RACE analysis using a reverse primer (GSP-*cadX*) designed to target *cadX*. Lines 2 and 4: 5′-RACE analysis using a reverse primer (NGSP-*cadX*) designed to target *cadX*. The amplification product indicated by the white arrow was subsequently purified, ligated into the pMD19T vector, and then sent for sequencing. (C) The sequence of the *cadDX* region in *S. suis* GZ0565. The double underline is the sequence including* the cadDX* operon promoter region in this study. The single underline is the sequence including the *cadX* promoter region in this study. The sequence marked in blue is the ORF of *cadD*. The sequence marked in green is the ORF of *cadX*. The single “G” with a red background is the additional TSS of *cadX.* (D) Analysis of the binding between CadX and its own promoter. (E) Activity of β-galactosidase under the control of the *cadX* promoter in the WT and Δ*cadDX *strains. Data are presented as mean ± SD, and “ns” indicate no significantly different values.

**Additional file 7. Differential gene expression in ΔcadDX compared with the WT.**

**Additional file 8. Validation of gene expression by RT-qPCR analysis.** Seven upregulated and six downregulated DEGs were selected to confirm the reliability of the transcriptome data.

**Additional file 9. Influence of the cadDX operon on the regulation of S. suis core genomic genes. **(A-D) The activities of β-galactosidase under the control of the promoters of *Fab*, *permease*, *PTS*, and *FeoA *in the WT and Δ*cadDX *strains. Data are presented as mean ± SD, and asterisks indicate significantly different values (“*”, “**”, and “***” indicate *p* < 0.05, *p* < 0.01, and *p* < 0.001, respectively). (E-H) CadX cannot bind to the promoter of *Fab*, *permease*, *PTS*, or *FeoA*.

**Additional file 10. Permease and FeoA** **are involved in antioxidative stress. **Survival rates of the WT, Δ*permease*, and OE-*FeoA *strains under H_2_O_2_ stress conditions. Data are presented as mean ± SD, and asterisks indicate significantly different values (“*” and “**” indicate *p* < 0.05 and *p* < 0.01, respectively).

**Additional file 11. Bacterial loads of S. agalactiae strains in mouse spleens.** Five mice per group were injected intraperitoneally with 1.0 × 10^2^ CFU of Δ*CRISPR*_*S.a*_-pSET2 or Δ*CRISPR*_*S.a*_-*cadDX*. All the mice were euthanized at 24 h post-infection. Bacteria from the spleen were plated onto THA, and colonies were expressed as log_10_CFU/g. Data are presented as mean ± SD, and “ns” indicates no significant difference.

## Data Availability

The transcriptome data from this study have been deposited in the NCBI SRA database under the accession numbers SRR28595173, SRR28595174, SRR28595175, and SRR28595176.
