# Supplementary material for: The cadDX operon contributes to cadmium resistance, oxidative stress resistance, and virulence in zoonotic streptococci
Source: Vet Res. 2024 Sep 27;55:119. doi: 10.1186/s13567-024-01371-1 (PMC11430099; doi:10.1186/s13567-024-01371-1)
Supplement: Supplementary file 1 — Additional file 1. Bacterial strains and plasmids used in this study. [file 13567_2024_1371_MOESM1_ESM.docx]

**Additional file 1. Bacterial strains and plasmids used in this study**

| Strains and plasmids | Characteristics or sequences (5’-3') | Sources or functions |
| --- | --- | --- |
| **Strains** | | |
| GZ0565 | *Streptococcus suis* serotype 9 virulent strain | Serotype 9 virulent strain |
| Δ*cadDX* | The deletion mutant of *cadDX* in the background of strain GZ0565 | This study |
| C-*cadDX* | *cadDX* complemented strain | This study |
| C-*cadD* | *cadD* complemented strain | This study |
| C-*cadX* | *cadX* complemented strain | This study |
| Δ*permease* | The deletion mutant of *permease* in the background of strain GZ0565 | This study |
| OE-*FeoA* | Overexpression of *FeoA* in the background of strain GZ0565 | This study |
| GD201008-001 | *Streptococcus agalactiae* virulent strain | Dong et al. [19] |
| *S. agalactiae-*pSET2 | pSET2 empty plasmid in the background of GD201008-001 | This study |
| *S. agalactiae-cadDX* | Expression of *cadDX* in the background of GD201008-001 | This study |
| *S. agalactiae*-*cadD* | Expression of *cadD* in the background of GD201008-001 | This study |
| *S. agalactiae*-*cadX* | Expression of *cadX* in the background of GD201008-001 | This study |
| Δ*CRISPR* | The *CRISPR* deletion mutant in the background of GD201008-001 | Dong et al. [19] |
| Δ*CRISPR-*pSET2 | pSET2 empty plasmid in the background of Δ*CRISPR* | This study |
| Δ*CRISPR*-*cadDX* | Expression of *cadDX* in the background of Δ*CRISPR* | This study |
| *E. coli* DH5α | For cloning and maintaining plasmids | TIANGEN |
| *E. coli* BL21 | For expressing plasmids | TIANGEN |
| **Plasmids** | | |
| pSET2 | For constructing complemented strains | Takamatsu et al. [18] |
| pSET2-*cadDX* | For *cadDX* complemented | This study |
| pSET2-*cadD* | For *cadD* complemented | This study |
| pSET2-*cadX* | For *cadX* complemented | This study |
| pMD19T | For gene cloning | Takara |
| pMD19T-P*cadXown* | For sequencing of *cadX* 5’ RACE | This study |
| pET28a | For protein expression | Novagen |
| pET28a-CadX | For CadX expression | This study |
| pTCV-*LacZ* | Reporter plasmid | Poyart et al. [29] |
| pTCV-P*cadDX*-*LacZ* | Reporter plasmid for *cadDX* promoter | This study |
| pTCV-P*cadXown*-*LacZ* | Reporter plasmid for *cadX* own promoter | This study |
| pTCV-P*PTS*-*LacZ* | Reporter plasmid for *PTS* promoter | This study |
| pTCV-P*Fab*-*LacZ* | Reporter plasmid for *Fab* promoter | This study |
| pTCV-P*FeoA*-*LacZ* | Reporter plasmid for *FeoA* promoter | This study |
| pTCV-P*permease*-*LacZ* | Reporter plasmid for *permease* promoter | This study |
