# Supplementary material for: The cadDX operon contributes to cadmium resistance, oxidative stress resistance, and virulence in zoonotic streptococci
Source: Vet Res. 2024 Sep 27;55:119. doi: 10.1186/s13567-024-01371-1 (PMC11430099; doi:10.1186/s13567-024-01371-1)
Supplement: Supplementary file 2 — Additional file 2. Primers used in this study. [file 13567_2024_1371_MOESM2_ESM.docx]

**Additional file 2. Primers used in this study**

| Primers | Sequences (5’-3') | Comment |
| --- | --- | --- |
| **Construction of strains** | | |
| P-1 | TTCTGAGGATGGTAAGGAGATAG | Detection of 11K MGE circularization |
| P-2 | ACTAACTACTCTACCTTTCACTC |  |
| P-3 | GGTAGAGTAAAAGGTAGAGTTCT |  |
| P-4 | GTCGTACTCACTATAAAGTAGCA |  |
| Δ*cadDX*-A | TGAAGGTCTGACAATTCTAAT | Upstream of fusion fragment for marked Δ*cadDX* |
| Δ*cadDX*-B | **AAGGAGTTTTCAGCATTATCC**TTAATCAACTAGGATAACAATCG |  |
| Δ*cadDX*-C | **ATATTCATTCTAATTGGTAATCAGATT**CTAAATAGATGTTAATGATGTCT | Downstream of fusion fragment for marked Δ*cadDX* |
| Δ*cadDX*-D | TTAGTCACAATGTCCGTCGTATC |  |
| Δ*cadDX*-A | TGAAGGTCTGACAATTCTAAT | Upstream of fusion fragment for unmarked Δ*cadDX* |
| Δ*cadDX*-B1 | **AGACATCATTAACATCTATTTAG**TTAATCAACTAGGATAACAATCG |  |
| Δ*cadDX*-C1 | CTAAATAGATGTTAATGATGTCT | Downstream of fusion fragment for unmarked Δ*cadDX* |
| Δ*cadDX*-D | TTAGTCACAATGTCCGTCGTATC |  |
| Δ*cadDX*-E | GAGCGATTGTTATCCTAGTT | Detection of deletion of *cadDX* gene |
| Δ*cadDX*-E | ACTGGAGATTACTCACGTTC |  |
| Δ*permease*-A | TGAAGTTAGCAAAAGTTTTAGCA | Upstream of fusion fragment for marked Δ*permease* |
| Δ*permease*-B | **AAGGAGTTTTCAGCATTATCC**AATTTCCCTCTTTCAACATATCA |  |
| Δ*permease*-C | **ATATTCATTCTAATTGGTAATCAGATT**GAGGAAATCATGATGTCTAAAAT | Downstream of fusion fragment for marked Δ*permease* |
| Δ*permease*-D | ACGCAATAACTACTCTACCTTTT |  |
| Δ*permease*-A | TGAAGTTAGCAAAAGTTTTAGCA | Upstream of fusion fragment for unmarked Δ*permease* |
| Δ*permease*-B1 | **ATTTTAGACATCATGATTTCCTC**AATTTCCCTCTTTCAACATATCA |  |
| Δ*permease*-C1 | GAGGAAATCATGATGTCTAAAAT | Downstream of fusion fragment for unmarked Δ*permease* |
| Δ*permease*-D | ACGCAATAACTACTCTACCTTTT |  |
| Δ*permease*-E | AAATGGAAACATTATAGCCTAAG | Detection of deletion of *permease* gene |
| Δ*permease*-F | AATAGTTTTCCTGCAGATACAAC |  |
| Δ*permease*-G | AATAATTGCGGTTTTATTGCTCT |  |
| Δ*permease*-H | CATTATTAGGAACGGTAACCCCA |  |
| *SacB-Spc*-F | GGATAATGCTGAAAACTCCTT | Sucrose sensitivity and spectinomycin resistance gene cassette *SacB*-*Spc* |
| *SacB-Spc*-R | AATCTGATTACCAATTAGAATGAATAT |  |
| pSET2-F | TAAGTTGGGTAACGCCAGGG | Detection of inserted fragments of pSET2 |
| pSET2-R | TTCCGGCTCGTATGTTGTGT |  |
| pSET2-*cadDX*-F | ccgGAATTCTCAATAAGAACTCTACCTTTTAC | Fragment for construction of C-*cadDX* |
| pSET2-*cadDX*-R | ccgGGATCCTATAGACAAAGGAACTGGAATAG |  |
| pSET2-*cadDX*-F | ccgGAATTCTCAATAAGAACTCTACCTTTTAC | Fragment for construction of C-*cadD* |
| pSET2-*cadD*-R | ccgGGATCCCTAGCCTAACACCGTCCATAGC |  |
| pSET2-*cadX*-F | ccgGGATCCTCAATAAGAACTCTACCTTTTAC | Upstream of fusion fragment for construction of C-*cadX* |
| pSET2-*cadX*-G | AAAAGACCTCATTCAAATATATT |  |
| pSET2-*cadX*-H | **AATATATTTGAATGAGGTCTTTT**ATAGCAGGTGGTTTATTGCC | Downstream of fusion ragment for construction of C-*cadX* |
| pSET2-*cadX*-R | ccgCTGCAGGAACTGGAATAGAGCGATTGTTA |  |
| pSET2-*FeoA*-F | CGGGGATCCTAGTTTTATTTGGGGATGACTTC | Fragment for construction of pSET2-*FeoA* |
| pSET2-*FeoA*-G | TGTGCTTCTATTATACCATATTG |  |
| pSET2-*FeoA*-H | **CAATATGGTATAATAGAAGCACA**ATGAAACTGCAAAACCTTC |  |
| pSET2-*FeoA*-R | CGGCTGCAGCTTATCGTCGTCATCCTTGTAATC TTTCCCCACCTCGCTT |  |
| pET28a-CadX-F | GCTACCTCCGCCACCACTTCCACCGCCTCCAGAACCTCCTCCACC TCCTAGTTGATTAAAAAAAT | Fusion fragment for construction of pET28a-CadX |
| pET28a-CadX-R | GGTGGAGGAGGTTCTGGAGGCGGTGGAAGTGGTGGCGGAGGTAGCTTGAAAAAAGATAGTATCTGCC |  |
| pTCV-P*cadDX*-*LacZ*-F | AACAAATGAATTCCCGGGTTTGGATGGCAGGGCTTTTT | Fusion fragment for construction of pTCV-P*cadDX*-*LacZ* |
| pTCV-P*cadDX*-*LacZ*-R | AAGCTGGGGATCCCCGGGAAAAGACCTCATTCAAATA |  |
| pTCV-P*cadXown*-*LacZ*-F | AACAAATGAATTCTATAGCAGGTGGTTTATTGCC | Fusion fragment for construction of pTCV-P*cadXown*-*LacZ* |
| pTCV-P*cadXown*-*LacZ*-R | AAGCTGGGGATCCAATATTTTCTCCTAGCCTAACAC |  |
| pTCV-P*PTS*-*LacZ*-F | AACAAATGAATTCCCGGGTCCTTCCTTTCCACAGCTT | Fusion fragment for construction of pTCV-P*PTS*-*LacZ* |
| pTCV-P*PTS*-*LacZ*-R | AAGCTGGGGATCCCCGGGTTCTTCCTCCGATGCAACC |  |
| pTCV-P*Fab*-*LacZ*-F | AACAAATGAATTCCCGGGCCATTGTAACACAGTTTTTAAAA | Fusion fragment for construction of pTCV-P*Fab*-*LacZ* |
| pTCV-P*Fab*-*LacZ*-F | AAGCTGGGGATCCCCGGGTTTCAAAATACAGTCAGTATTT |  |
| pTCV-P*FeoA*-*LacZ*-F | AACAAATGAATTCCCGGGTGCTAGTTGCCGGGATTGAA | Fusion fragment for construction of pTCV-P*FeoA*-*LacZ* |
| pTCV-P*FeoA*-*LacZ*R | AAGCTGGGGATCCCCGGGTAGTATAGCACAATTCCTTAAAAG |  |
| pTCV-P*permease*-*LacZ*-F | AACAAATGAATTCCCGGGTGTTTCTCTCCTTTAATGAT | Fusion fragment for construction of pTCV-P*permease*-*LacZ* |
| pTCV-P*permease*-*LacZ*-R | AAGCTGGGGATCCCCGGGACAACCACCTCATATCAAAA |  |
| pTCV-JD-F | GTTGAATAACACTTATTCCTATC | Detection for construction of pTCV-*LacZ* |
| pTCV-JD-R | CTTCCACAGTAGTTCACCACC |  |
| **5’ RACE** | | |
| GSP-*cadX* | CGAAGATGATGGGAGGCAGATGCCATAC | Primers for 5’ RACE of *cadX* |
| NGSP-*cadX* | CCTTTTCAAGGTAGTTCGTTGCGGTTG |  |
| pMD19T-F | GAGCGGATAACAATTTCACACAGG | Detection of 5’ RACE fragment of *cadX* inserted to pMD19T |
| pMD19T-R | CGCCAGGGTTTTCCCAGTCACGAC |  |
| **EMSA** | | |
| EMSA-*cadDX*-F | TTTGGATGGCAGGGCTTTTT | The promoter fragment of *cadDX* |
| EMSA-*cadDX*-R | AAAAGACCTCATTCAAATA |  |
| *16S*-F | AAAAGGCTGTGGCTTAACCATAG | The fragment of *16S rRNA* |
| *16S*-R | GGTATCTAATCCTGTTCGCTCCC |  |
| EMSA-*PTS*-F | TCCTTCCTTTCCACAGCTT | The promoter fragment of *BFP66_RS02205* (*PTS*) |
| EMSA-*PTS*-R | TTCTTCCTCCGATGCAACC |  |
| EMSA-*Fab*-F | CCATTGTAACACAGTTTTTAAAA | The promoter fragment of *BFP66_RS08355* (*Fab*) |
| EMSA-*Fab*-R | TTTCAAAATACAGTCAGTATTT |  |
| EMSA-*FeoA*-F | TGCTAGTTGCCGGGATTGAA | The promoter fragment of *BFP66_RS02660* (*FeoA*) |
| EMSA-*FeoA*-R | TAGTATAGCACAATTCCTTAAAAG |  |
| EMSA-*permease*-F | TGTTTCTCTCCTTTAATGAT | The promoter fragment of *BFP66_RS01320* (*permease*) |
| EMSA-*permease*-R | ACAACCACCTCATATCAAAA |  |
| EMSA-*cadXown*-F | TATAGCAGGTGGTTTATTGCC | The fragment of *cadX* own promoter |
| EMSA-*cadXown*-R | AATATTTTCTCCTAGCCTAACAC |  |
| **RT-qPCR** |  |  |
| *qparC*-F | GGTAGCACAGTGGGTTCTTT | The transcriptional level of *RS05620* (*parC*) mRNA |
| *qparC*-R | GTCGGAAATGGCTGGTTATCT |  |
| *qcadX*-F | CAAGATGTTTTGATTGGCTA | The transcriptional level of *RS01345* (*cadX*) mRNA |
| *qcadX*-R | TTTTATCAAAGTTTACCGAT |  |
| *qcadD*-F | CAAAAGTAATCATAGCGACT | The transcriptional level of *RS01350* (*cadD*) mRNA |
| *qcadD*-R | TTAGGACAATTTCTAGGCTC |  |
| *qadhP*-F | CATACCACCGTCAACAGAGTAG | The transcriptional level of *RS01370* (*adhP*) mRNA |
| *qadhP*-R | TAAGGTTCCTGGTCGCATTC |  |
| *qadhE*-F | GTTGACCCAGCACTCGTATT | The transcriptional level of *RS01375* (*adhE*) mRNA |
| *qadhE*-R | TTCCAAAGTAGCCTTCAACC |  |
| *qglpF*-F | GCCCAATAAACCACTCACAAAG | The transcriptional level of *RS05750* (*glpF*) mRNA |
| *qglpF*-R | AGCAGGAGTAGTGCTAGATAAGA |  |
| *qglpO*-F | GAACCTTCTCCACCCAGATTAC | The transcriptional level of *RS05755* (*glpO*) mRNA |
| *qglpO*-R | GGTATCGTCGCTCGTGATTT |  |
| *qglpK*-F | CGGAAGGCCTGTTTCCTTAT | The transcriptional level of *RS05760* (*glpK*) mRNA |
| *qglpK*-R | CTGTGATTGCAGGGTCCTTTA |  |
| *qarcD*-F | ATGGATCCCGTGTGAAGTAAAG | The transcriptional level of *RS06190* (*arcD*) mRNA |
| *qarcD*-R | CCAGAAATCCCTGCTGAAGAA |  |
| *qarcC*-F | TAGAGTGGCTATTCGGGTTAGA | The transcriptional level of *RS06185* (*arcC*) mRNA |
| *qarcC*-R | CCAGAATGATACGGAAGGAAGAG |  |
| *qarcB*-F | GTTACGACCATCACCACAGTAA | The transcriptional level of *RS06180* (*arcB*) mRNA |
| *qarcB*-R | CATGGCACCCAACTCAAATG |  |
| *qM6_Spy1295*-F | CTACACCGGTCAAGACGATAAA | The transcriptional level of *RS06175* (*M6_Spy1295*) mRNA |
| *qM6_Spy1295*-R | GAGGTATTCCAGTTGTCCAAGA |  |
| *qarcA*-F | GCGCGATAGCACAAATCAAAG | The transcriptional level of *RS06170* (*arcA*) mRNA |
| *qarcA*-R | GCAGGGATTGTAGTCGGTTTAG |  |
| *qfabH*-F | GCCTACAGTAGTCAAACCTTCTT | The transcriptional level of *RS08345* (*fabH*) mRNA |
| *qfabH*-R | GATTTGGACGCAGATTCATTGG |  |
| *qfabT*-F | CCCTCGTCGCAAATTCAAATATAG | The transcriptional level of *RS08350* (*fabT*) mRNA |
| *qfabT*-R | CTAGTCTGACAGCAGGGATAGA |  |
| *qfabM*-F | CTCTGCATCATCCATACCATCT | The transcriptional level of *RS08355* (*fabM*) mRNA |
| *qfabM*-R | GCGAGTTGTTCATCTCTACCT |  |

^a^ Bolded nucleotides denote reverse complement; underlined nucleotides denote restriction enzyme sites
