# Supplementary figures and images for: The cadDX operon contributes to cadmium resistance, oxidative stress resistance, and virulence in zoonotic streptococci
Source: Vet Res. 2024 Sep 27;55:119. doi: 10.1186/s13567-024-01371-1 (PMC11430099; doi:10.1186/s13567-024-01371-1)

**
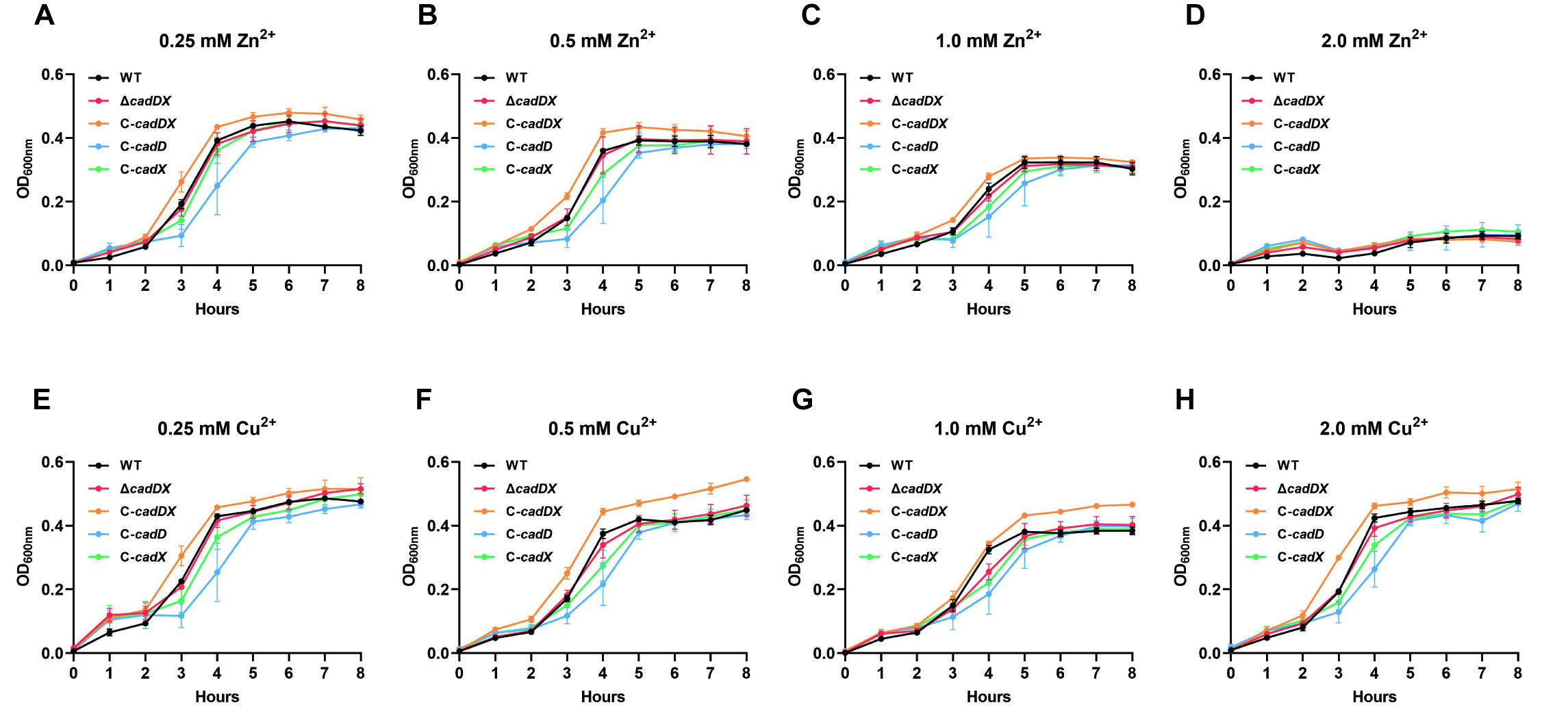
**

Supplement: Supplementary file 5 — Additional file 5. WT, ΔcadDX, C-cadDX, C-cadD, and C-cadX respond to zinc and copper stress. (A-D) Growth curves of the WT, ΔcadDX, C-cadDX, C-cadD, and C-cadX strains in THB supplemented with various concentrations of ZnCl2 (0.25, 0.5, 1.0, and 2.0 mM). (E-H) Growth curves of the WT, ΔcadDX, C-cadDX, C-cadD, and C-cadX strains in THB supplemented with various concentrations of CuSO4 (0.25, 0.5, 1.0, and 2.0 mM). [file 13567_2024_1371_MOESM5_ESM.docx]

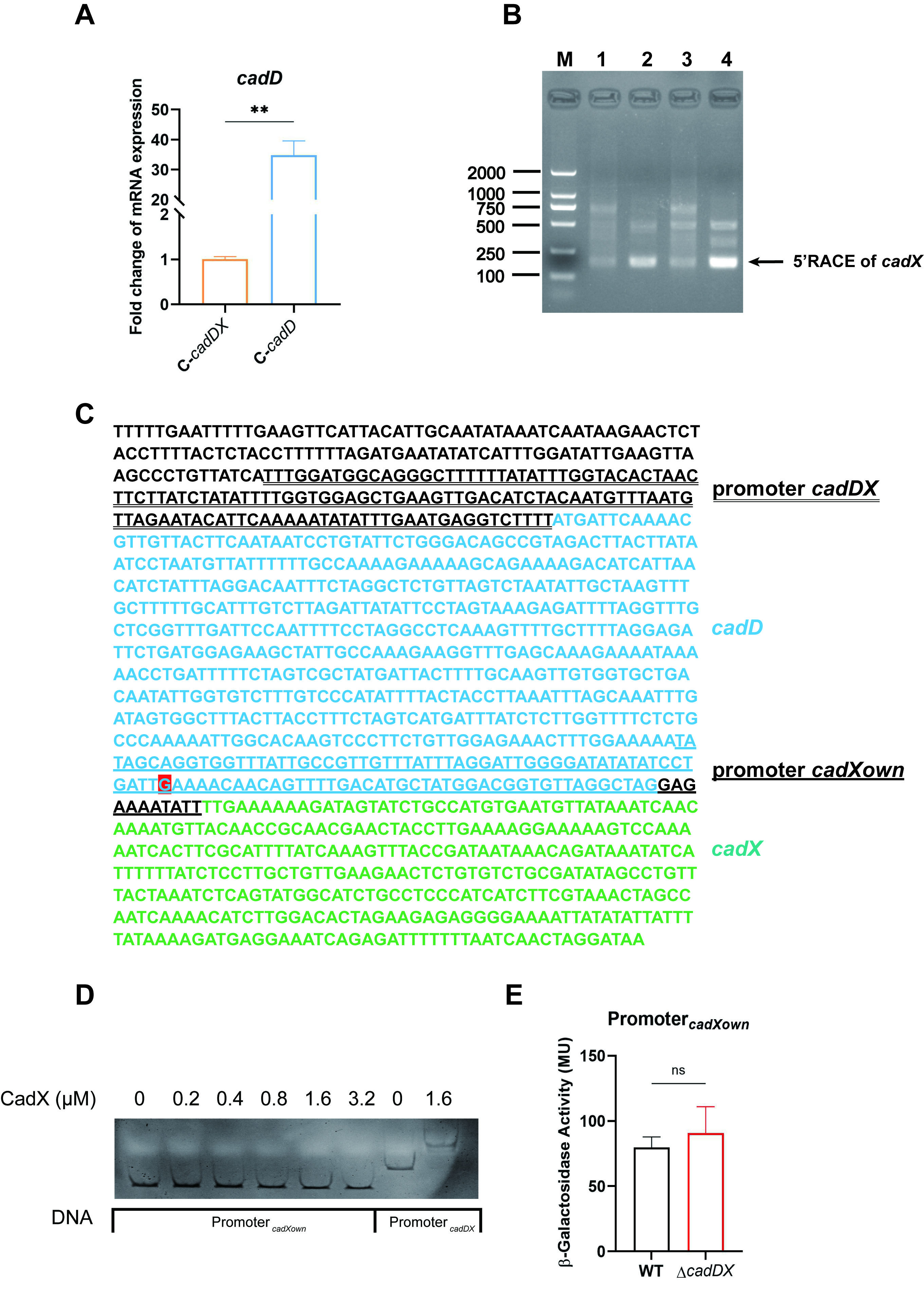

Supplement: Supplementary file 6 — Additional file 6. cadX possesses its own promoter. (A) Expression of cadD in C-cadDX and C-cadD. Data are presented as mean ± SD, and asterisks indicate significantly different values (“**” indicates p < 0.01). (B) M: Molecular weight markers. Lines 1 and 3: 5′-RACE analysis using a reverse primer (GSP-cadX) designed to target cadX. Lines 2 and 4: 5′-RACE analysis using a reverse primer (NGSP-cadX) designed to target cadX. The amplification product indicated by the white arrow was subsequently purified, ligated into the pMD19T vector, and then sent for sequencing. (C) The sequence of the cadDX region in S. suis GZ0565. The double underline is the sequence including the cadDX operon promoter region in this study. The single underline is the sequence including the cadX promoter region in this study. The sequence marked in blue is the ORF of cadD. The sequence marked in green is the ORF of cadX. The single “G” with a red background is the additional TSS of cadX. (D) Analysis of the binding between CadX and its own promoter. (E) Activity of β-galactosidase under the control of the cadX promoter in the WT and ΔcadDX strains. Data are presented as mean ± SD, and “ns” indicate no significantly different values. [file 13567_2024_1371_MOESM6_ESM.docx]

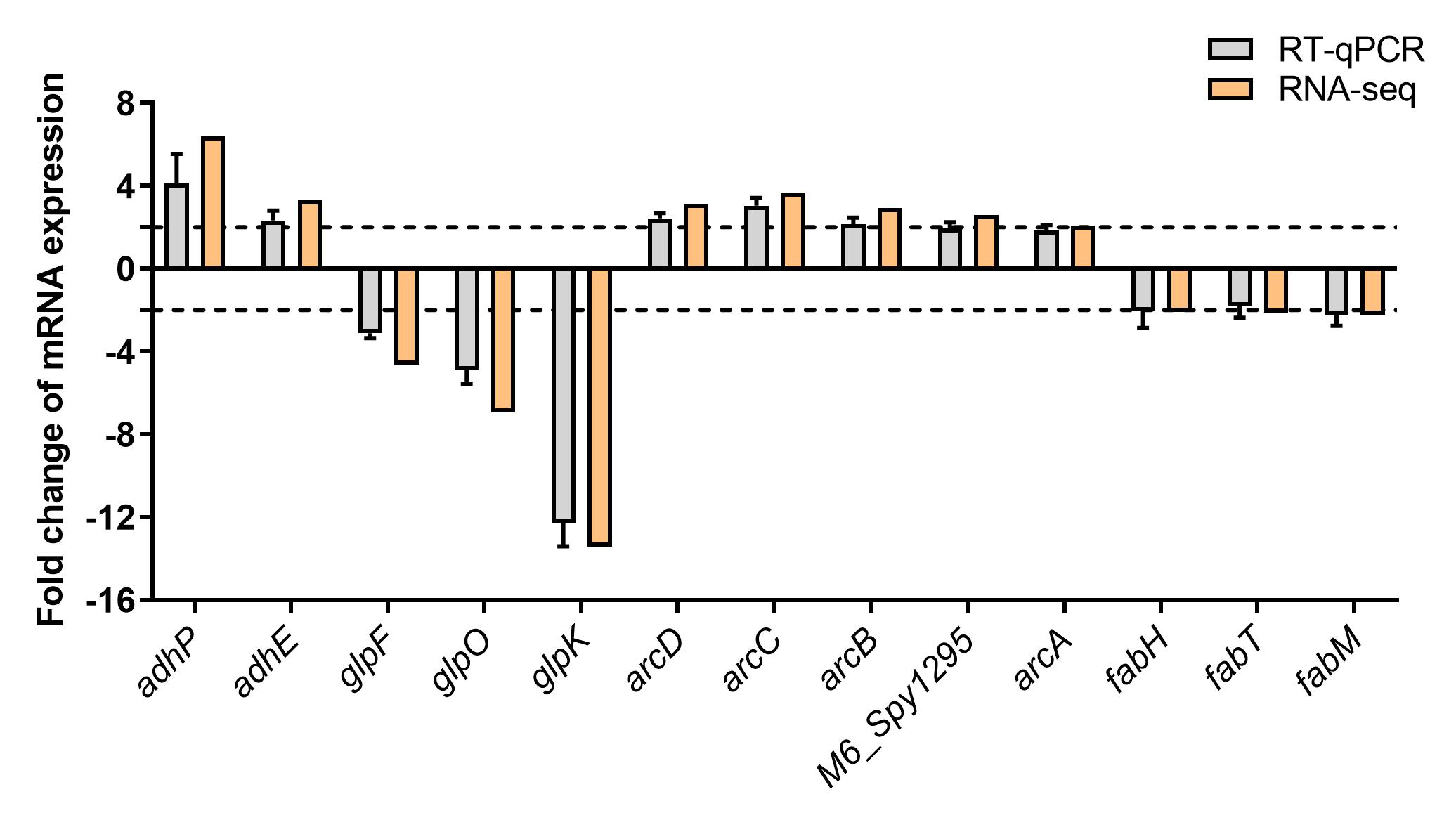

Supplement: Supplementary file 8 — Additional file 8. Validation of gene expression by RT-qPCR analysis. Seven upregulated and six downregulated DEGs were selected to confirm the reliability of the transcriptome data. [file 13567_2024_1371_MOESM8_ESM.docx]

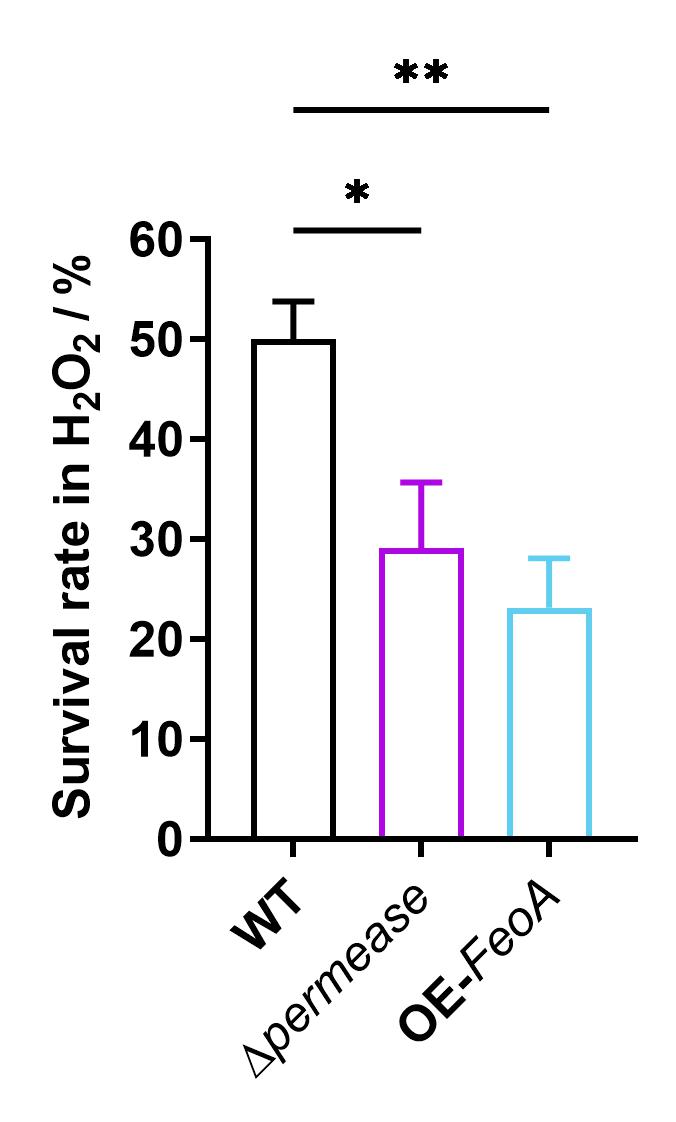

Supplement: Supplementary file 9 — Additional file 9. Influence of the cadDX operon on the regulation of S. suis core genomic genes. (A-D) The activities of β-galactosidase under the control of the promoters of Fab, permease, PTS, and FeoA in the WT and ΔcadDX strains. Data are presented as mean ± SD, and asterisks indicate significantly different values (“*”, “**”, and “***” indicate p < 0.05, p < 0.01, and p < 0.001, respectively). (E-H) CadX cannot bind to the promoter of Fab, permease, PTS, or FeoA. [file 13567_2024_1371_MOESM9_ESM.docx]

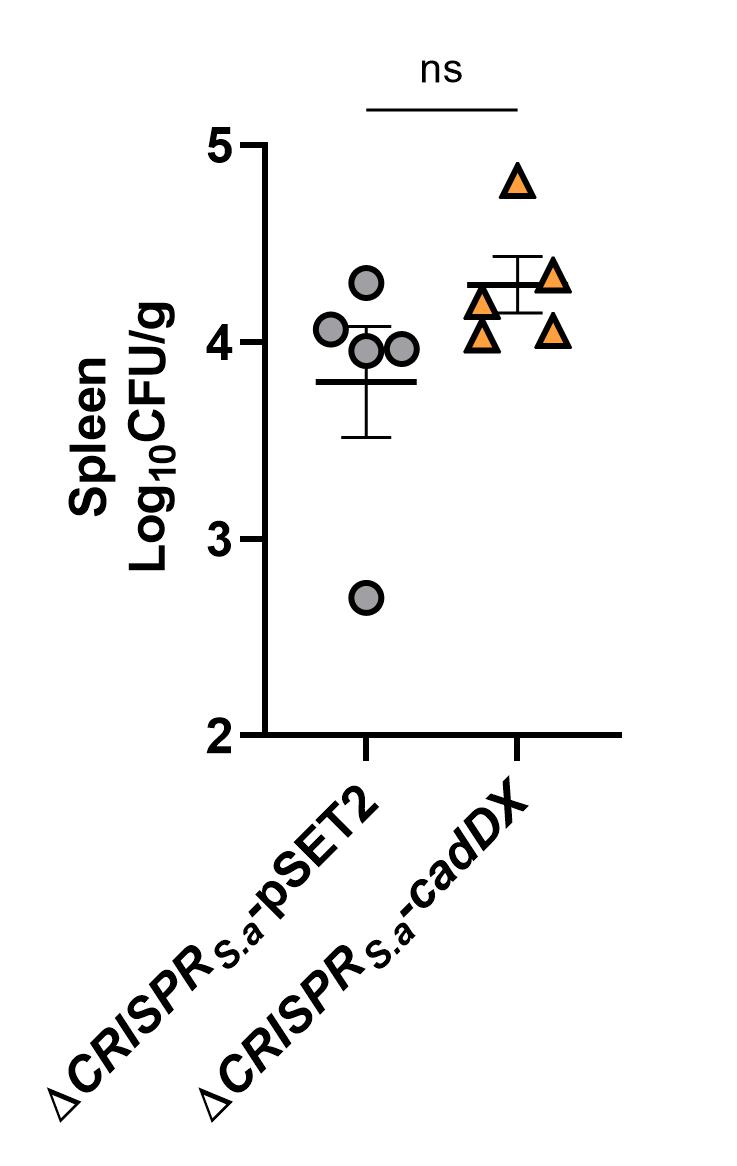

Supplement: Supplementary file 10 — Additional file 10. Permease and FeoA are involved in antioxidative stress. Survival rates of the WT, Δpermease, and OE-FeoA strains under H2O2 stress conditions. Data are presented as mean ± SD, and asterisks indicate significantly different values (“*” and “**” indicate p < 0.05 and p < 0.01, respectively). [file 13567_2024_1371_MOESM10_ESM.docx]
