# Supplementary material for: The cadDX operon contributes to cadmium resistance, oxidative stress resistance, and virulence in zoonotic streptococci
Source: Vet Res. 2024 Sep 27;55:119. doi: 10.1186/s13567-024-01371-1 (PMC11430099; doi:10.1186/s13567-024-01371-1)
Supplement: Supplementary file 7 — Additional file 7. Differential gene expression in ΔcadDX compared with the WT. [file 13567_2024_1371_MOESM7_ESM.docx]

**Additional file 7. Differential gene expressions in Δ*cadDX* compared to the WT**

| ID | log_2_ (FC) | *p* Value | Description |
| --- | --- | --- | --- |
| *BFP66_RS00750* | -1.48911 | 0.02819 | single-stranded DNA-binding protein |
| *BFP66_RS01325* | -1.13064 | 0.008676 | permease |
| *BFP66_RS01340* | 4.837192 | 3.02E-06 | site-specific integrase |
| *BFP66_RS01370* | 2.676507 | 4.63E-09 | alcohol dehydrogenase AdhP |
| *BFP66_RS01375* | 1.729785 | 4.46E-09 | bifunctional acetaldehyde-CoA/alcohol dehydrogenase AdhE |
| *BFP66_RS01800* | 1.290835 | 0.001013 | galactokinase |
| *BFP66_RS01805* | 1.267066 | 0.000615 | UDP-glucose--hexose-1-phosphate uridylyltransferase |
| *BFP66_RS02205* | 1.639172 | 5.94E-08 | beta-galactosidase |
| *BFP66_RS02210* | 1.812019 | 5.1E-07 | PTS system mannose/fructose/N-acetylgalactosamine-transporter subunit IIB |
| *BFP66_RS02215* | 1.721139 | 8.5E-07 | PTS mannose/fructose/sorbose/N-acetylgalactosamine transporter subunit IIC |
| *BFP66_RS02220* | 1.772361 | 1.25E-06 | PTS system mannose/fructose/sorbose family transporter subunit IID |
| *BFP66_RS02225* | 1.694751 | 7.77E-06 | PTS fructose transporter subunit IIA |
| *BFP66_RS02230* | 1.635428 | 0.004171 | galactose mutarotase |
| *BFP66_RS02660* | 1.262031 | 0.01426 | ferrous iron transport protein A FeoA |
| *BFP66_RS02690* | 2.00481 | 4.43E-05 | N-acetylmannosamine-6-phosphate 2-epimerase |
| *BFP66_RS02695* | 1.083536 | 0.000465 | Phosphotransferase system IIC component, glucose/maltose/N-acetylglucosamine-specific |
| *BFP66_RS02960* | -1.04454 | 0.006138 | cation-translocating P-type ATPase |
| *BFP66_RS03555* | 1.475532 | 0.000343 | gluconate 5-dehydrogenase |
| *BFP66_RS03560* | 1.1556 | 0.031153 | RpiB/LacA/LacB family sugar-phosphate isomerase |
| *BFP66_RS03565* | 1.303801 | 0.003778 | sugar kinase |
| *BFP66_RS03580* | 1.144262 | 0.001176 | glycosyl hydrolase family 88 |
| *BFP66_RS03585* | 1.206451 | 0.030039 | PTS system mannose/fructose/N-acetylgalactosamine-transporter subunit IIB |
| *BFP66_RS03590* | 1.382715 | 4.15E-05 | PTS mannose/fructose/sorbose/N-acetylgalactosamine transporter subunit IIC |
| *BFP66_RS03595* | 1.306666 | 0.000164 | PTS mannose/fructose/sorbose transporter family subunit IID |
| *BFP66_RS03600* | 1.004405 | 0.018739 | preprotein translocase subunit YajC |
| *BFP66_RS04080* | -2.1041 | 0.008081 | Uncharacterised protein |
| *BFP66_RS04315* | 10.46659 | 0.017071 | PTS lactose/cellobiose transporter subunit IIA |
| *BFP66_RS04450* | 2.731511 | 0.015893 | CAAX amino terminal protease family protein |
| *BFP66_RS04975* | -2.91561 | 0.047395 | replicon stabilization protein |
| *BFP66_RS05055* | -1.10125 | 0.000426 | hypothetical protein A6M16_05165 |
| *BFP66_RS05260* | -1.03421 | 0.007547 | GMP reductase |
| *BFP66_RS05570* | -2.02704 | 0.009544 | membrane protein |
| *BFP66_RS05750* | -2.2098 | 2.44E-10 | aquaporin family protein GlpF |
| *BFP66_RS05755* | -2.79585 | 1.71E-19 | type 1 glycerol-3-phosphate oxidase GlpO |
| *BFP66_RS05760* | -3.74788 | 1.64E-41 | glycerol kinase GlpK |
| *BFP66_RS06170* | 1.642673 | 2.85E-09 | YfcC family protein arcD |
| *BFP66_RS06175* | 1.874783 | 8.02E-10 | carbamate kinase arcC |
| *BFP66_RS06180* | 1.557257 | 2.54E-09 | ornithine carbamoyltransferase arcB |
| *BFP66_RS06185* | 1.371521 | 1.36E-06 | GNAT family N-acetyltransferase M6_Spy1295 |
| *BFP66_RS06190* | 1.050362 | 1.92E-05 | arginine deiminase arcA |
| *BFP66_RS06520* | 1.139042 | 1.34E-05 | N-carbamoylputrescine amidase |
| *BFP66_RS06530* | 1.215406 | 5.07E-06 | carboxynorspermidine decarboxylase |
| *BFP66_RS06535* | 1.212559 | 2.3E-06 | saccharopine dehydrogenase family protein |
| *BFP66_RS06540* | 1.253964 | 8.91E-07 | spermidine synthase |
| *BFP66_RS06545* | 1.037853 | 0.000033 | aminotransferase class I/II-fold pyridoxal phosphate-dependent enzyme |
| *BFP66_RS06675* | 1.321575 | 0.032493 | hypothetical protein A6M16_06775 |
| *BFP66_RS07050* | 1.177506 | 0.000404 | alpha-galactosidase |
| *BFP66_RS07445* | -1.34425 | 0.020569 | DUF3272 domain-containing protein |
| *BFP66_RS07520* | -3.66297 | 0.012717 | transposase, ISSmi2 |
| *BFP66_RS08285* | -1.4129 | 0.000118 | CPBP family intramembrane metalloprotease |
| *BFP66_RS08290* | -1.04412 | 0.003156 | GNAT family N-acetyltransferase |
| *BFP66_RS08345* | -1.05071 | 0.000786 | ketoacyl-ACP synthase III FabH |
| *BFP66_RS08350* | -1.09303 | 2.61E-05 | MarR family transcriptional regulator FabT |
| *BFP66_RS08355* | -1.14067 | 0.001076 | enoyl-CoA hydratase FabM |
| *BFP66_RS08500* | 1.093978 | 0.01504 | histidine phosphatase family protein |
| *BFP66_RS08510* | 1.133437 | 0.000929 | PTS cellobiose transporter subunit IIC |
| *BFP66_RS08520* | 1.456742 | 0.002507 | lactose-specific phosphotransferase system (PTS), IIA component 1 |
| *BFP66_RS08820* | -2.67286 | 6.48E-06 | bifunctional glycosyltransferase family 2/GtrA family protein |
| *BFP66_RS08825* | -1.92515 | 0.00517 | phosphodiester glycosidase family protein |
| *BFP66_RS08830* | -4.61651 | 1.56E-06 | hypothetical protein A6M16_08935 |
| *BFP66_RS08960* | 1.381871 | 0.01007 | extracellular solute-binding protein |
| *BFP66_RS08970* | 2.525461 | 0.023665 | sugar ABC transporter permease |
| *BFP66_RS09535* | 1.00937 | 0.008529 | argininosuccinate synthase |
| *BFP66_RS09690* | -1.33212 | 0.034993 | hypothetical protein |
| *BFP66_RS10065* | -2.71567 | 0.040866 | hypothetical protein EXW74_04055 |
